# New cyclopentaquinoline and 3,5-dichlorobenzoic acid hybrids with neuroprotection against oxidative stress for the treatment of Alzheimer’s disease

**DOI:** 10.1080/14756366.2022.2158822

**Published:** 2023-01-11

**Authors:** Kamila Czarnecka, Małgorzata Girek, Paweł Kręcisz, Robert Skibiński, Kamil Łątka, Jakub Jończyk, Marek Bajda, Piotr Szymczyk, Grzegorz Galita, Jacek Kabziński, Ireneusz Majsterek, Alba Espargaró, Raimon Sabate, Paweł Szymański

**Affiliations:** a Department of Pharmaceutical Chemistry, Drug Analyses and Radiopharmacy, Faculty of Pharmacy, Medical University of Lodz, Lodz, Poland; b Department of Medicinal Chemistry, Faculty of Pharmacy, Medical University of Lublin, Lublin, Poland; c Department of Physicochemical Drug Analysis, Chair of Pharmaceutical Chemistry, Faculty of Pharmacy, Jagiellonian University Medical College, Krakow, Poland; d Department of Biology and Pharmaceutical Botany,Faculty of Pharmacy, Medical University of Lodz, Lodz, Poland; e Department of Clinical Chemistry and Biochemistry, Medical University of Lodz, Lodz, Poland; f Department of Pharmacy and Pharmaceutical Technology and Physical Chemistry, Faculty of Pharmacy and Food Sciences, University of Barcelona, Barcelona, Spain; g Institute of Nanoscience and Nanotechnology (IN2UB), Barcelona, Spain; h Department of Radiobiology and Radiation Protection, Military Institute of Hygiene and Epidemiology, Warsaw, Poland

**Keywords:** acetylcholinesterase inhibitors, Alzheimer’s disease, molecular modelling, Ellman’s method, MTDL

## Abstract

Alzheimer’s disease (AD) is a progressive neurodegenerative brain disease. Thus, drugs including donepezil, rivastigmine, and galantamine are not entirely effective in the treatment of this multifactorial disease. The present study evaluates eight derivatives (**3a**–**3h**) as candidates with stronger anti-AD potential but with less side effects. Reactive oxygen species (ROS) assays were used to assess oxidative stress which involve in the neurodegeneration. The neuroprotective properties of 3e against oxidative stress were done in three experiments using MTT test. The anti-AD potential was determined based on their anticholinesterase inhibition ability, determined using Ellman’s method, Aβ aggregation potential according to thioflavin (Th) fluorescence assay, and their antioxidative and anti-inflammatory activities. Compound **3e** exhibited moderate cholinesterase inhibition activity (AChE, IC_50_ = 0.131 µM; BuChE, IC_50_ = 0.116 µM; SI = 1.13), significant inhibition of Aβ(1–42) aggregation (55.7%, at 5 µM) and acceptable neuroprotective activity. Extensive analysis of in vitro and in vivo assays indicates that new cyclopentaquinoline derivatives offer promise as candidates for new anti-AD drugs.

## Introduction

Alzheimer’s disease (AD) is progressive neurodegenerative disorder and the most common cause of dementia in modern society. AD is characterised by progressive cognitive dysfunctions, often accompanied by aggression or depression. A key hallmark in AD pathogenesis which can exacerbate its progression is oxidative stress, because the brain is most vulnerable to oxidative stress. In the early stages of AD are seen oxidative modifications in cerebral tissue^1^. The aetiology of AD has not been exactly explained, but several factors are characteristic of the development of disease: the deposition of amyloid β (Aβ) plagues, hyperphosphorylation of microtubule associated protein tau (MAPT), oxidative stress, inflammation process, dyshomeostasis of biometals and a reduction in the level of acetylcholine (ACh) caused by damage to the cholinergic system^2–7^. Much evidence has shown a link between the major pathological processes of Alzheimer’s disease and oxidative stress^8^. Aβ plagues are formed from amyloid β peptides cleaved by beta and gamma secretases from amyloid precursor protein (APP). Aβ itself is formed from 39 to 42 amino acid residues. The most common isoforms of Aβ are Aβ40 and Aβ42^9^; the former has a higher ability to form fibrils than the latter and also induces stronger neurotoxity^2^^,^^4^. The pathology of the tau protein (MAPT) is one of the characteristic factors of AD^10^. MAPT binds to microtubules and supports cell skeleton^11^. The presence of an imbalance between kinase and phosphatase activity results in hyperphosphorylation of MAPT, resulting in cytoskeleton and microtubule dysfunctions and improper axonal transport^12^. Hyperphosphorylated tau protein takes part in the formation of neurofibrillary tangles, which disturb neuronal function and cause the death of neurons^13^.

Current approaches in AD pharmacotherapy are based on cholinergic hypothesis, and hence aim to increase the level of ACh. This can be achieved by influencing the activity of cholinesterase (ChE). There are two enzymes, which play a key role in hydrolysing ACh – acetylcholinesterase (AChE) and butyrylcholinesterase (BuChE). AChE is more active than BuChE in a heathy brain but not in the case of AD: the activity of AChE remains unchanged or slightly lower, but BuChE may increase^14^^,^^15^. The vast majority of modern AD pharmaceuticals are AChE inhibitors; however, a new approach addresses the inhibition of both enzymes, partly to restore the balance, but also because both take part in Aβ aggregation^16^^,^^17^. In addition, AChE can form an AChE-Aβ complex, which is more neurotoxic than Aβ alone^18^. The first reversible AChE and BuChE inhibitor used in AD therapy was tacrine (9-amino-1,2,3,4-tetrahydroacridine, THA), but it was withdrawn from market due to hepatotoxicity. This fact has not prevented research on tetrahydroacridine derivatives, such as cylopentaquinoline, partly because these structures demonstrate good activity towards AChE and partly because they have lower molecular masses, which are associated with favourable pharmacokinetics in AD therapy. The free amine group present in the THA molecule is believed to account for its hepatotoxicity; however, it is also a good point for substitution with a new molecule, thus decreasing hepatotoxicity and allowing the design of multi-target-directed ligands (MTDLs)^19^^,^^20^. MTDLs are compounds designed to simultaneously approach several biological targets to improve therapy^21^. Due to the complex nature of AD, MTDLs currently represent the best potential solution for AD therapy^22^, and hence their design has generated considerable research interest^23–25^.

Inflammatory reaction is a part of the immune system response, whose aim is to remove harmful stimuli (irritants, damaged cells or pathogens). Inflammation is part of the innate immunity system, and can be divided into acute and chronic inflammation. Chronic inflammation comprises long-term processes that result from the failure to eliminate the causes of acute inflammation, autoimmune responses or chronic irritant of low intensity. It is associated with the development of chronic diseases such as AD, cancers or rheumatoid arthritis. Several cellular enzymes, of which hyaluronidase plays an important role, control the inflammatory process. Hyaluronidase decreases the integrity of tissues during inflammation through depolymerisation of hyaluronan. Nowadays, the non-steroidal anti-inflammatory drugs (NSAIDs) are commonly used to treat inflammatory processes; however, their use should be limited due to side effects. Therefore, new drugs with additional anti-inflammatory properties are being synthesised and tested^26^^,^^27^.

**Figure 1.**
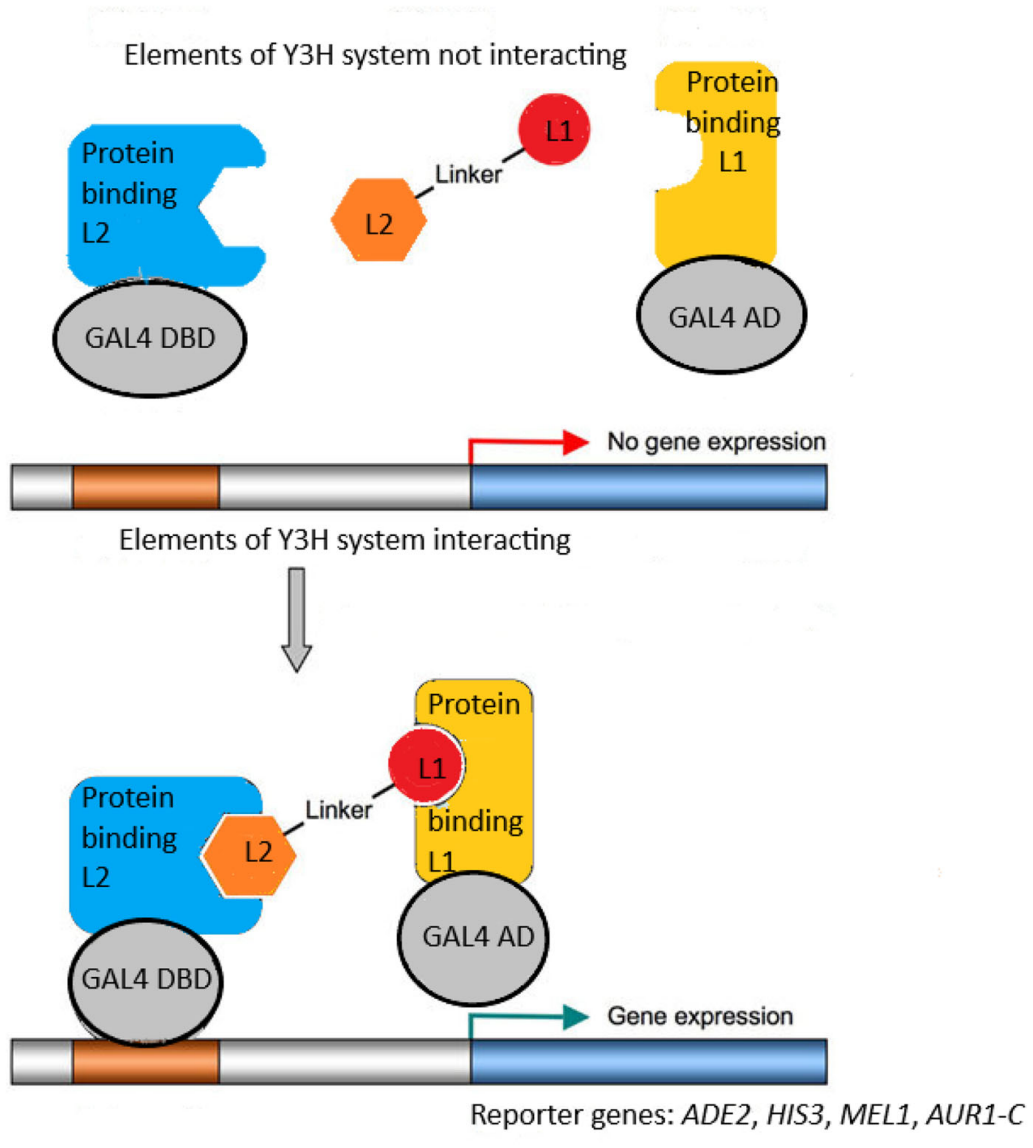
The idea of yeast three-hybrid (Y3H) system [31]. Components: I. Hybrid ligand composed of ligand 1 (L1), linker and ligand 2 (L2) II. First hybrid protein comprised of protein binding L1 and activating domain (AD) of yeast GAL4 transcription factor. III. Second hybrid protein comprised of protein binding L2 and DNA-binding domain (DBD) of yeast GAL4 transcription factor.

Yeast three-hybrid technology (Y3H) (Figure 1) is based on the two-hybrid system (Y2H). The Y2H methodology depends on the interaction between two hybrid proteins, activating the expression of reporter genes, resulting in the growth of yeast on selective media^28^^,^^29^. Y2H uses a hybrid ligand molecule to mediate protein-protein interaction. Y3H technology, in contrast, is widely used to identify new small molecules interacting with known receptors, or to confirm known ligand-receptor interactions^30–33^. The present study uses the Y3H method to evaluate in vivo the hybrid ligand-mediated interactions between human acetylcholinesterase and four proteins: human amyloid beta A4 (A4), human beta-secretase 1 A (BACE-1A), human monoamine oxidase B (MAO B) and human microtubule associated protein tau (MAPT).

Currently we have advanced computational biology tools to identify effective targets in AD therapy. Thanks to the possibility of using *in silico* research, it is possible to quickly determine the effectiveness of the new compounds. It needs to be highlighted that computational studies are predictive in nature. Further *in vitro* and *in vivo* tests are needed to confirm the mechanism of action^34^^,^^35^. The purpose of the in vivo experiment was to evaluate new cyclopentaquinoline derivatives according to the OECD 423 “Acute Oral Toxicity - Acute Toxic Class Method”. Such classification of tetrahydroacridine derivatives helps determine the doses that can be used in further pharmacokinetics and behavioural studies on animals, thus avoiding unforeseen situations caused by the potential toxicity of chemical substances after their administration to animals. Cyclopentaquinoline and tetahydroacridine derivatives are obtained by the chemical modification of tacrine, effective drug that was used in AD but was withdrawn due to its strong side effects. The new derivatives are characterised by better efficacy and less toxicity *in vitro*.

Therefore, following on from our previous research regarding the development of anti-Alzheimer’s agents, the aim of the present study was to synthesise and evaluate new cyclopentaquinoline derivatives with 3,5-dichlorobenzoic acid. In earlier publication by Czarnecka et al, we obtained tetrahydroacridine derivatives with good properties. The obtained results for 3,5-dichlorobenzoic acid derivatives inspired us to expand the syntheses and the scope of research for these promising hybrids. In this way, developed this group of molecules (presented in this publication) and design new structures cyclopentaquinoline that have never been published^36^. We elaborated new therapeutic compounds against ROS-mediated damage in AD. New inhibitor exerts a neuroprotective effect via antioxidative and anti-inflammatory activities. The new derivatives were designed with the aim of acting as potential AChE and BuChE inhibitors with significant additional properties. The study employs biological assays to determine the potential for AChE/BuChE inhibition, Aβ aggregation inhibition. It also employs molecular modelling, physicochemical assays of logP and pKa, and ADMET computer prediction using experimental logP and pKa values.

## Materials and methods

### Chemistry

All solvents, chemicals and reagents were obtained commercially and used without purification. Thin layer chromatography (TLC) was used to monitor the reactions. All melting points were determined in open glass capillaries and are uncorrected. ^1^H spectra were recorded using CD_3_OD or DMSO-d6 as a solvent on a Brucker Advance III 600 MHz spectrometer with tetramethylsilane as an internal standard. Chemical shifts (δ) are given in parts per million (ppm) and spin multiplicities are given as s (singlet), d (doublet), t (triplet), q (quartet) or m (multiplet). Coupling constants (J) are expressed in hertz (Hz). IR spectra were recorded on attenuated total reflectance (ATR) mode (Thermo Scientific Nicolet 6700) in ATR. Fourier transformation was performed using an infra-red spectrometer with smart ITR diamond adapter (Madison, Wisconsin USA). High resolution mass spectra (HRMS) analysis was performed with the use of Agilent Accurate-Mass Q-TOF LC/MS G6520B system with dual electrospray (DESI) source (Agilent Technologies, Santa Clara, USA). The detector was used in positive mode with an Agilent ESI-L tuning mix in high resolution mode (4 GHz). Purification by flash chromatography was carried out on silica gel 60 (Merck). Intermediates **1a**–**1h** were prepared according to the previously literature method^37^.

#### General procedure for the synthesis of compounds 2a–2h

A mixture of 10 ml tetrahydrofuran (THF), 3,5-dichlorobenzoic acid (0.04 g, 0.19 mM) was maintained at −5 °C with stirring. Next, N-methylomorpholine (0.02 ml, 0.19 mM) was added dropwise to the solution. The mixture was stirred. After 2 h a solution of **1a** (0.042 g, 0.19 mM), **1 b** (0.044 g, 0.19 mM), **1c** (0.047 g, 0.19 mM), **1d** (0.050 g, 0.19 mM), **1e** (0.052 g, 0.19 mM), **1f** (0.055 g, 0.19 mM), **1 g** (0.057 g, 0.19 mM) or **1 h** (0.060 g, 0.19 mM) (dissolved in 5 ml THF) were added. All reactions have been continuing for 24 h. Then, the reaction was filtrated, solvent was removed under reduced pressure and the crude product was purified by normal phase adsorption flash chromatography to give the desired product. Details about synthesis procedures of compounds **2a**-**2h** and MS spectra were described in Supplementary materials.

#### General procedure for the synthesis of compounds 3a–3h

Compounds **2a** (0.020 g, 0.050 mM), **2 b** (0.020 g, 0.048 mM), **2c** (0.020 g, 0.047 mM), **2d** (0.020 g, 0.045 mM), **2e** (0.020 g, 0.044 mM), **2f** (0.020 g, 0.043 mM), **2 g** (0.020 g, 0.041 mM) and **2 h** (0.020 g, 0.040 mM), were dissolved in methanol (1 ml). Then, 4 ml of HCl/ether was added to the mixtures and the reaction was stirred for 10 min. After 24 h, precipitates were formed, isolated by filtration and dried. In this synthesis **3a**–**3h** compounds were obtained physical and spectral data are listed below. Details about synthesis procedures of compounds **3a**–**3h** and MS spectra were described in Supplementary materials.

### Biological evaluation

#### In vitro inhibition studies on AChE and BuChE

The capacity of the novel cyclopentaquinoline hybrids to inhibit AChE (from the electric eel) and BuChE (from equine serum) were determined by the modified colorimetric Ellman’s method^38^^,^^39^. Both enzymes, DTNB – Ellman’s reagent (5,5′-dithiobis-(2-nitrobenzoic acid)), acetyltiocholine iodide (ATCI) were obtained from Sigma Aldrich. AChE and BuChE samples in phosphate buffer (PBS) pH 8.0 were incubated in 96-well plates in the presence of the substrate (ATCI), 76 µl of DTNB and the tested compound at various concentration at room temperature for 10 min (AChE) or eight minutes (BuChE). The production of the yellow anion was measured at a wavelength of 412 nm. The concentration of each compound needed to inhibit 50% of enzyme activity (IC_50_) was obtained as the mean ± SD of three independent experiments performed in triplicate.

#### Neuroprotection against oxidative stress

##### Cell culture

The SH-SY5Y (human neuroblastoma) (European Collection of Cell Culture) was chosen to determine the neuroprotective properties of the novel compounds. The Ham’s F12:EMEM (1:1) medium was used (Sigma Aldrich), comprising 15% Foetal Bovine Serum (Biowest), 2 mM Glutamine (Sigma Aldrich), 100 units/ml penicillin and 100 mg/ml streptomycin (Biological Industries) and 1% Non-Essential Amino Acids (Biological Industries).

##### MTT assays

SH-SY5Y cells were seeded at a density of 5 × 10^3^ cells/well in 96-well plates. The cells were cultured for 24 h (37 °C, 5% CO_2_). After 24 h of incubation, the medium was removed and cells were exposed to 100 µl of the compound at a range of concentrations, or of culture medium alone (blank control). After incubation, the medium was removed, washed with PBS and 50 µl of the MTT solution was added. The plates were then incubated for two hours (37 °C, 5% CO_2_). The MTT solution was then removed from the wells and 100 µl of DMSO was added. The plates were incubated for 10 min at room temperature and then 5 µl of Sorensen Buffer was added. The plates were swayed and the absorbance measured in a microplate reader (Synergy H1, BioTek, Winooski, VT, USA) at a wavelength of 570 nm. Cell viability was expressed as a percentage of control values (blank control). The experiments were done in triplicate^40^.

##### Neuroprotection

The neuroprotective properties of **3e** against oxidative stress were done in three experiments. First experiment regarded use of hydrogen peroxide (H_2_O_2_) to generate exogenous free radicals. Cells were incubated with compounds in the range of concentrations (0.01–10 µM) for 24 h. Then, H_2_O_2_ (100 µM) was added to the cells, and the incubation in the presence of compounds were carried out for next 24 h. In the second and third experiments, mixture of rotenone (30 µM) and oligomycin A (10 µM) (R/O) was used to induce mitochondrial reactive oxygen species. In the second experiment, cells were incubated with compound **3e** in the range of concentration before the addition of R/O for 24 h. After incubation, R/O mixture was added and cells were incubated with **3e** for additional 24 h. In the third experiment, cells were cultured for 24 h without compound **3e** and without R/O mixture. After first incubation, R/O mixture and compound **3e** were added at the same time and the incubation took 24 h. Trolox was used as a positive control. Each experiment was done three times. Cell death was measured by the MTT test. Data were shown as the percentage of the reduction of MTT in regard to non-incubated cells^39^^,^^41^^,^^42^.

#### Hyaluronidase inhibition test

Hyaluronidase inhibition was established by turbidimetry according to USP XXII-NF XVII^43^, which was further adapted to use in 96-well plates by Piwowarski et al.^44^ and the principle of this assay is based on the following reaction:

$$\mathrm{hyaluronic}\;\mathrm{acid}\overset{\mathrm{hyaluronidase}}{\to }\;\mathrm{depolymerization}\;\mathrm{products}+\mathrm{hyaluronic}\;\mathrm{acid}$$

The ability to inhibit hyaluronidase was assessed using a turbidimetric method based on spectrophotometric measurement of turbidity resulting from the formation of hyaluronan-bovine albumin complexes in an acidic environment. The analysis was performed against a positive control. Briefly, 20 µl of the tested compound in monosodium phosphate buffer (pH 7.0) was added to 40 µl of hyaluronidase solution (22.55 U/ml, Sigma Aldrich) in the same buffer. The mixture was incubated for 10 min in the dark at 37 °C. Next, 40 µl of hyaluronic acid solution (0.03%, Sigma Aldrich) in monosodium phosphate buffer (pH 5.35) was added to all wells and the mixture was further kept in the dark at 37 °C for 45 min. Finally, 300 µl of bovine serum albumin solution (0.1%, Serva) in sodium acetate buffer (pH 3.75) was added to the mixture, and the 96-well plate was incubated at room temperature for next 10 min. Changes in turbidity at 600 nm were measured by a microplate reader (BioTek, Winooski, VT, USA). Heparin (WZF, Polfa) was used as a positive control. For the investigated compound, the test was run in triplicates in three experiments to calculate IC50 values^45^.

The inhibitory activity of the tested compound against hyaluronidase was calculated following the Equation (1):

(1) $$\text{\% inhibition}=100\;\times \;(1-(\frac{A_{\mathrm{HA}}-A_{\mathrm{AN}}}{A_{\mathrm{HA}}-A_{\mathrm{HYAL}}})),$$

where A_HA_ − absorbance of solution without the enzyme (positive control), A_HYAL_ − absorbance of solution without the tested compound (negative control), A_AN_ − absorbance of solution with the tested compound.

#### Cell culture and cytotoxicity assay

Human hepatic stellate cells (HSCs, Sciencell) were grown in the Stellate Cell Medium (Sciencell) supplemented with 2% Foetal Bovine Serum (Sciencell), 1% Stellate Cell Growth Supplement (Sciencell) and 1% penicillin/streptomycin solution (Sciencell). The cells were cultured in the incubator at 37 °C and 5% CO_2_. To start the experiment, the cells were seeded at a density of 5 × 103 cells/well in 96-well plates and incubated (37 °C, 5% CO_2_) for 24 h. After incubation, medium was removed from the wells and the cells were exposed to 100 µl of the compound over a range of concentrations (from 0.1 to 10 µM) or 100 µl of culture medium alone (blank control). The cells were then further incubated for 24 h. After this time, the medium was removed, the cells were washed with PBS and 50 µl of MTT solution (0.75 mg/ml) was added to each well. The plates were incubated in the dark at 37 °C for another two hours. The MTT solution was then removed from the wells and 100 µl of DMSO was added. The plates were incubated at room temperature for 10 min and then 5 µl of Sorensen Buffer was added to each well. The plates were swayed and the absorbance at 570 nm was measured with a microplate reader (Synergy H1, BioTek, Winooski, VT, USA). The cell viability was expressed as a percentage of the control values (blank control)^40^^,^^46^^,^^47^.

#### In vivo acute oral toxicity

Compound **3e** was administered by the oral route (by gavage) in one of four fixed doses (5, 50, 300, 2000 mg/kg) for a given stage of the study. The results allow to classify the compound according to the Globally Harmonised Classification System for Chemical Substances and Mixtures (GHS). In addition, the LD_50_ cut-off values was determined. According to the figure from the OECD 423 guidelines, a maximum of six test stages can be performed. Three animals (female mice, Balb/c) took part in each stage, and the number of stages depends on the mortality of the animals, which is the final test parameter. The research was carried out on the basis of the agreement of the Local Ethical Committee for animal testing in Łódź No. 56/115 ŁB/2018.

After quarantine, handling, weighing and labelling, the compound **3e** was given in a dose of 300 mg/kg (300 mg/kg is a dose used in the absence of information on the toxicity of the test substance. If 2–3 animals die after using this dose, compound will be given at a lower dose − 50 mg/kg. Last dose 5 mg/kg is used if after a dose of 50 mg/kg also 2–3 animals die. If no death occurs, or death of one animal, the compound is again administered to the stomach in the same dose 300 mg/kg, and in the case of subsequent deaths, a dose of 2000 mg/kg of body weight is used). The presence of a substance-dependent mortality of animals exposed at one stage determines the performance of the next stage of the study. After each dose, animals are observed for 14 days. After the observation period, the animals are euthanized by intraperitoneal administration of a lethal dose of pentobarbital sodium and subjected to a post-mortem examination. All organs, which were found with macroscopic changes, were taken for histopathological examination^48^.

#### Y3H assay

The Y3H method was used to analyse the possible hybrid-ligand induced interactions between five selected proteins. Constructs containing ACHE, A4, BACE-1A, MAO B and MAPT were prepared by Gene Universal Inc. (Newark, DE, USA), all of which had been cloned into pGBKT7 (ACHE) or pGADT7 (A4, BACE1A, MAO B, MAPT) vectors. Yeast codon optimisation was performed to facilitate high expression of human recombinant proteins in *Saccharomyces cerevisae*. The fragments for the signal peptide (aa 1–51) and domain responsible for protein tetramerization (aa 578–611) were obtained from the cDNA encoding for human ACHE (GenBank M55040.1). Therefore, the prepared pGBKT7-hACHE plasmid contained the cDNA fragment encoding for aa 52–577. The pGADT7-hA4 construct was obtained from the human amyloid A4 protein fragment (UniProtKB-P05067-1), encoding for the 42 aa protein (aa 672–713); the pGADT7-hBACE1A plasmid was obtained from the extracellular, N-terminal domain (aa 46–457) of BACE1A (GenBank: AF204943.1); the pGADT7-hBACE1A plasmid incorporated the fragments encoding for the signal peptide (aa 1–21), propeptide (aa 22–45), transmembrane (aa 458–478) and cytoplasmic (aa 479–501) domains; the pGADT7-hMAPT plasmid included the cDNA encoding for the entire protein (aa 1–758) (UniProtKB-P10636-1); the pGADT7-hMAO B plasmid was obtained from the cDNA encoding for the cytoplasmic domain (aa 1–489) of human MAO B.

All plasmids contain inserts cloned in a correct frame as NdeI/BamHI fragments. The prepared plasmids were used to transform competent *S. cerevisiae* cells as described in the Matchmaker Gold yeast two-hybrid system manual (Takara/Clontech, USA). The pGBKT7-hACHE plasmid was used to transform Y2HGold strain, while the other four plasmids were used to transform the Y187 strain. All negative and positive controls described in the manual of Matchmaker Gold yeast two-hybrid system were performed. The transformed yeast strain Y2HGold [pGBKT7-hACHE] was tested for bait autoactivation.

To exclude the potential protein-protein interactions between bait (ACHE) and four prey (A4, BACE1A, MAO B, MAPT) a small scale mating procedure (5 ml) was performed according to Matchmaker Gold yeast two-hybrid system manual recommendations. Prepared cell suspension was plated on DDO agar plates containing aureobasidin A (200 ng/ml) and X-α-Gal (40 µg/ml) (DDO/X/A) to screen for potential protein-protein interactions. A small scale mating procedure (10 ml) was then performed in the presence of hybrid ligands (10 µM) to initiate the possible hybrid ligand-mediated protein interactions. A suspension of mated cells was plated on DDO agar plates containing 10 µM of hybrid -ligand, aureobasidin A (200 ng/ml) and X-α- Gal (40 µg/ml) (QDO/X/A). Blue colonies were transferred on the QDO/X/A agar plates containing hybrid ligand (10 µM) confirm the presence of interactions. Obtained blue colonies were transferred on QDO/X/A agar plates. No hybrid ligand was added to the QDO/X/A agar to confirm that the interaction is dependent on hybrid ligand.

The yeast ẞ-galactosidase microplate assay protocol (stopped) was used to evaluate the ẞ-galactosidase activity in the yeast culture according to the manufacturer’s instructions. The appropriate kit was supplied by Thermo Fischer Scientific (USA). The lack of protein-protein interaction between ACHE and BACE-1 was excluded in control tests. The yeast cells used in the assay were grown for 96 h (30^0^C) on liquid QDO medium, containing 150 μg/ml aureobasidin A, 10 μM of ligand and 40 μg/ml X-α-Gal. Optical density was measured at 660 nm and absorbance at 420 nm by a Synergy H1 spectrophotometer (Biotek). In the quantitative ẞ-galactosidase assay, the hybrid ligand **3e** was used. The ẞ-galactosidase assay was realised in triplicate.

#### In vitro β -amyloid assay

A promising approach for developing effective therapeutics for AD involves the identification of agents that inhibit the aggregation of Aβ42, as Aβ(1–42) has a high propensity to form fibrils in the brain and cause serious neurotoxicity. In the present study, the potential of the target compound to prevent self-mediated Aβ(1–42) aggregation was determined using Thioflavin T (ThT) fluorescence assay. Briefly, the peptide was incubated at room temperature for 24 h (final Aβ concentration 12.58 µM) with and without potential inhibitor compound (5, 10, 25, 50, 100 µM). After incubation, samples were diluted to a final volume of 100 µl with 20 µl phosphate buffered saline (PBS at pH 8.0) containing 5 µM of ThT. After a five-minute incubation with the dye, the fluorescence intensity was determined: emission was measured at 446 nm and excitation at 490 nm. The fluorescence intensities were compared and the % inhibition was calculated.

#### In cellulo β-amyloid assay

Inhibition assay in Escherichia coli cells over-expressing amyloid proteins. Cloning and over-expression of amyloid-prone sequences. *Escherichia coli* competent cells BL21 (DE3) were transformed with the required pET vectors carrying the DNA sequence of Aβ42 peptide. Because of the addition of the initiation codon ATG in front of genes, the over-expressed Aβ42 contains an additional methionine residue at their N terminus. For overnight culture preparation, a colony of BL21 (DE3) bearing the plasmid to be expressed was inoculated in 10 ml of M9 minimal medium containing 50 μg/ml of kanamycin at 37 °C. For the expression of Aβ42, the required volume of overnight culture to obtain a 1:500 dilution was added to fresh M9 minimal medium containing 50 μg/ml of kanamycin and 250 μM Thioflavin-S (Th-S). The bacterial culture was then grown at 37 °C, with orbital agitation of 250 rpm. When the cell density reached OD_600nm_–0.6, 980 μL of the culture were transferred into 1.5 ml conical tubes containing 10 μL of the compound to be tested dissolved in DMSO, and 10 μL of 100 mM isopropyl 1-thio-β-D-galactopyranoside (IPTG). The final concentration of the compound was set to 10 μM. The samples were grown overnight at 37 °C under agitation at 1400 rpm, using a Thermomixer (Eppendorf, Hamburg, Germany). As negative control (corresponding maximum amyloid presence), free DMSO was used. Concurrently, non-induced samples (i.e. without IPTG) were prepared and used as positive controls (absence of amyloid). These samples were also used to assess the potential intrinsic toxicity of the compounds and to confirm the correct bacterial growth^49^^,^^50^.

##### Th-S steady-state fluorescence determination

For ThS fluorescence determination, Th-S spectra were recorded on an Aminco Bowman series 2 luminescence spectrophotometer (Aminco-Bowman AB2, SLM Aminco, Rochester, NY, USA), from 460 to 600 nm at 25 °C, using an excitation wavelength of 445 nm and slit widths of 4 nm. The emission at 485 nm (Th-S fluorescence peak observed in the presence of amyloids) was recorded. To normalise the Th-S fluorescence as a function of the bacterial concentration in the in-cellulo assays, OD_600nm_ was determined using a Shimadzu UV-2401 PC UV − Vis spectrophotometer (Shimadzu, Japan). The fluorescence normalisation was carried out considering as 100% the Th-S fluorescence of the bacterial cells expressing Aβ42 in the absence of drug, and 0% the Th-S fluorescence of the bacterial cells non-expressing Aβ42 (in absence of the protein production inductor, IPTG). The obtain results are the average of duplicates of 10 independent experiments (average of 20 determinations). As internal control we used DP128, known anti-Aβ and tau drug^51^^,^^52^ obtaining inhibition capacity of 69.2 ± 4.1 for Aβ42 at 10 μM.

#### pKa assay

Potassium dihydrogen phosphate, potassium hydroxide and methanol (POCH) were used to prepare the buffer solution, as described previously^39^. The buffer pH value was set from 5.6 to 12.4 in steps of 0.2. The pH was measured in 23 °C by using a Mettler Toledo FiveEasy pH metre with a LE438 Lab pH electrode (Mettler Toledo). Our tested compound solution was 5 µM solution in methanol: water (1:1) mix.

Spectrophotometric measurement was performed in a 96-well plate using a Synergy H1 microplate reader (BioTek) with Gen5 software (BioTek). The full assay consisted of 35 UV spectrum measurements, one for each work buffer solution. For the assay, 180 μL sequent work buffer and 20 μL tested compound solution were added to each of 35 wells. For the blank, 200 μL sequent work buffer alone was added to each of 35 wells. The measurement was performed at 23 °C. The spectra were recorded from 280 nm to 380 nm in 1 nm steps. Concentrations were calculated based on the 332/343 nm and 343/332 nm ratio as described previously^39^. The results were compared with computer calculations of pKa value obtained using chemicalize.com online software (ChemAxon 2018) and ACD/Percepta version 14.0.0 (Advanced Chemistry Development, Inc., Metropolitan Toronto, Canada).

#### LogP assay

Methanol (POCH) was used as an organic modifier. Demineralised water was purified in our faculty. The methanol and water solution were set at pH 11 using 30 mM Triethylamine (TEA) (Sigma Aldrich). Ten isocratic mobile phases were used in the calibration and assay. The first mobile phase contained 50% methanol solution with 30 mM TEA and 50% water solution with 30 mM TEA. Each subsequent mobile phase contained 5% more methanol solution: the final mobile phase consisted of 95% methanol solution and 5% water solution mix. The method required uncharged substances to be used during analysis. Basic solutions were used, because the test compound and the six compounds used in the calibration were also basic; the basic properties of mobile phases did not affect the retention times of neutral calibration compounds.

The compounds used to prepare the calibration curve are given in Table 1. These substances were selected on the basis of their structural similarity to the test compounds. The stock solutions contained about 1 mg/ml of calibration compounds or test compounds. The injection concentration was 100 µg/ml, and the injection volume was 7 µl.

**Table 1.** Substances used to perform calibration curve.

Substance name	Experimental properties	
	logP^53^	pKa
Procainamide HCl	0.88	9.32^54^
Tacrine	2.71	9.95^55^
Thymol	3.30	–
Naphthalene	3.60	–
Promazine HCl	4.55	9.36^54^
Promethazine HCl	4.81	9.10^54^
Chlorpromazine HCl	5.41	9.30^53^
Thioridazine HCl	5.90	9.50^56^

A Waters 600 HPLC system was used with a photodiode array detector (PDA). The detector was set at the respective optimum absorption wavelength for each compound. The system used an Xbridge C18 50 mm × 4.6 mm i.d., 3.5 µm, chromatographic column (Waters). Data acquisition and processing were performed on Waters Millennium software.

The assay was performed according to Chao Liang et al. with modification^57^. To prepare the calibration curve each substance was eluted by all mobile phases. All obtained retention times were used for the calculations as described previously^39^. The results were obtained in the form of calibration curves and log_kw_ values of the test compound.

Test compound was eluted by all mobile phases. Retention times were used in mathematical equations to calculate the logkw value. The logP value was read from the calibration curve. The result was compared with computer calculations of the logP value performed by chemicalize.com online software (ChemAxon 2018) and ACD/Percepta version 14.0.0 (Advanced Chemistry Development, Inc., Metropolitan Toronto, Canada).

#### Docking studies

Corina online (Molecular Networks and Altamira) was used to create three-dimensional structures of compounds, that were then prepared using Sybyl 8.0 (Tripos). Protonation states were inspected, hydrogen atoms were added, atom types were checked and Gesteiger-Marsili charges were assigned. All ligands were docked to acetylcholinesterase from 2CKM and to butyrylcholinesterase based on a 1P0I crystal structure using GoldSuite 5.1 (CCDC). Before docking, the proteins were prepared in the following way: all histidine residues were protonated at Nε, the hydrogen atoms were added, ligand and water molecules were removed; the binding site was defined as all amino acid residues within 10 Å from bis-(7)-tacrine for AChE, and 20 Å from the glycerol molecule for BuChE. A standard set of genetic algorithms with a population size of 100, number of operations 100 000, and clustering with a tolerance of 1 Å was applied. After docking process, 10 ligand poses, sorted by GoldScore (for AChE) and ChemScore (for BuChE) were obtained. The results were visualised by PyMOL 0.99rc6 (DeLano Scientific LLC).

#### Molecular dynamics simulation

Docking pose of compound **2e** within acetylcholinesterase was used to build the system for molecular dynamics simulation. System builder implemented in Schrodinger Suite was utilised to solvate the complex with TIP3P water. Then, system was neutralised and 0.15 M sodium chloride was added. The 50 ns MD simulation in NPT ensemble, preceded by standard system minimisation and equilibration, was being run with Desmond. The results were analysed with Schrodinger Suite 2021-3 and VMD 1.9.3.

## Results

### Synthesis

**Scheme 1.**
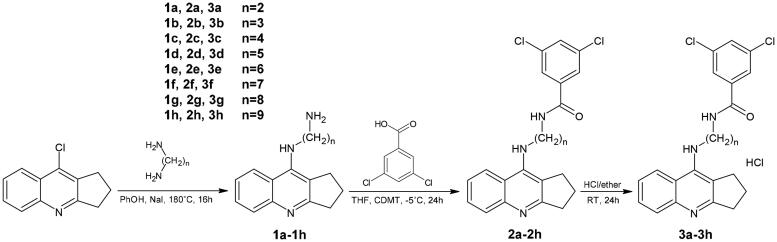
Synthesis of compounds **2a**–**2h** and **3a**–**3h**. Reagents: (a) 3,5-dichlorobenzoic acid, 2-Chloro-4,6-dimethoxy-1,3,5-triazine (CDMT), N-methylmorpholine, THF; (b) HCl/ether.

The synthesis of the designed compounds was carried out in two steps and is outlined in Scheme 1. The novel, multifunctional derivatives consisted of 2,3-dihydro-1H-cyclopenta[b]quinoline with 3,5-dichlorobenzoic acid moiety with an alkyl chain of varying lengths (*n* = 2–9). The first step used intermediates **1a**–**1h**, based on reactions described previously. Compounds **2a**–**2h** were dissolved in a small volume of methanol and then HCl in ether was added.

### Biological evaluation

#### In vitro inhibition studies on AChE and BuChE

To get the optimal linker length, new cyclopentaquinoline derivatives (**3a**–**3h**), tacrine and donepezil were evaluated for ChE inhibitory activity using modified Ellman’s method. The IC_50_ values for AChE and BuChE inhibitions are summarised in Table 2. Most of the new synthesised compounds showed high inhibitory potency against ChEs, indicating that the introduction of 3,5-dichlobenzoic acid not lower the inhibitory capacity of the target compounds. As shown in Table 2, compound **3e** (AChE, IC_50_ = 131 nM; BuChE, IC_50_ = 116 nM) was the best inhibitor against AChE and BuChE, revealing that a six-carbon linker between 3,5-dichlorobenzoic acid moiety and cyclopentaquinoline group was the suitable length for ChE inhibition. Derivative **3e** showed similar AChE inhibition values to the reference compounds (tacrine and donepezil), but was a better BuChE inhibitor compared to donepezil.

**Table 2.** The activity of novel compounds **3a**–**3h** against acetylcholinesterase from electric eel and equine butyrylcholinesterase.

Compound	AChE IC_50_ ± SD (µM)^a^	BuChE IC_50_ ± SD (µM)^b^	Selectivity for AChE^c^	Selectivity for BuChE^d^
**3a**	1.545 ± 0.121	0.225 ± 0.021	0.15	6.88
**3b**	1.179 ± 0.134	0.889 ± 0.027	0.56	1.78
**3c**	1.155 ± 0.130	0.126 ± 0.007	0.11	9.18
**3d**	0.211 ± 0.031	1.123 ± 0.180	5.33	0.19
**3e**	0.131 ± 0.016	0.116 ± 0.006	0.89	1.13
**3f**	0.253 ± 0.042	0.117 ± 0.027	0.46	2.16
**3g**	1.485 ± 0.133	0.330 ± 0.034	0.22	4.50
**3h**	0.145 ± 0.013	0.131 ± 0.011	0.91	1.10
Tacrine	0.118 ± 0.016	0.016 ± 0.002	0.14	7.38
Donepezil	0.123 ± 0.008	5.869 ± 0.379	47.72	0.02

^a^Inhibitor concentration (means ± SD of three experiments) for 50% inactivation of AChE. ^b^Inhibitor concentration (means ± SD of three experiments) for 50% inactivation of BuChE. ^c^Selectivity for AChE is defined as IC_50_(BuChE)/IC_50_(AChE). ^d^Selectivity for BuChE is defined as IC_50_(AChE)/IC_50_(BuChE).

#### Neuroprotection against oxidative stress

The neuroprotective potential of **3e** against oxidative stress was checked in three experiments. The first experiment examined whether **3e** acted against exogenous free radicals induced by H_2_O_2_ at concentrations of 10 µM, 1 µM, 0.1 µM and 0.01 µM, with Trolox used as a positive control (Table 3). **3e** was found to possess neuroprotective activity at concentrations of 1 µM, 0.1 µM and 0.01 µM; however, its effect was lower than for Trolox. The highest neuroprotective activity was obtained at a concentration of 0.1 µM, which is comparable with the IC_50_ of AChE inhibition assay (0.131 µM). In the one-way ANOVA, values of *p* ≤ 0.05 were considered statistically significant for **3e**, not for Trolox.

**Table 3.** SH-SY5Y cell viability (%) after treatment with H_2_O_2_ (100 µM) or mixture of rotenone (30 µM) and oligomycin A (10 µM) at selected concentrations of **3e**.

			Concentrations			
	Cells without compounds	Compound	10 µM	1 µM	0.1 µM	0.01 µM
H_2_O_2_ 100 µM	% cell viability					
	84.27 ± 0.03	**3e**	41.11 ± 3.60	95.44 ± 3.09	96.75 ± 4.23	90.57 ± 7.65
		Trolox	99.22 ± 3.30	99.38 ± 9.81	98.62 ± 1.36	96.41 ± 1.51
			% neuroprotection			
		**3e**		71	79.34	40.04
		Trolox	95.05	96.03	91.22	77.16
			0.1 µM	0.01 µM	0.001 µM	0.0001 µM
Rotenone/Oligomycin A 30/10 µM	% cell viability					
	46.77 ± 2.17	Pre-incubation **3e**	48.05 ± 1.73	50.84 ± 7.02	53.40 ± 7.03	56.15 ± 6.55
		Pre-incubation Trolox	44.26 ± 3.73	46.38 ± 2.06	47.91 ± 4.48	46.23 ± 3.65
	47.91 ± 4.02	Co-incubation **3e**	46.76 ± 6.52	49.38 ± 4.59	52.43 ± 6.60	54.55 ± 6.72
		Co-incubation Trolox	48.09 ± 8.01	50.34 ± 8.11	50.83 ± 6.98	42.11 ± 6.28
			% neuroprotection			
		Pre-incubation **3e**	2.41	7.65	12.46	17.64
		Pre-incubation Trolox	–	–	2.15	–
		Co-incubation **3e**	–	2.82	8.67	12.75
		Co-incubation Trolox	0.35	4.67	5.60	–

Cell viability was determined by the MTT test. The results were expressed as mean ± SD. The experiment was performed in quadruplicate and repeated three times.

R/O mixture was used to induce ROS production in mitochondria by inhibiting the mitochondrial electron transport chain – complexes I and V^58^. A pre-incubation assay was performed to test whether **3e** exerted neuroprotective activity by the activation of endogenous antioxidant pathways. A co-incubation test was performed to check whether the compound was a free-radical scavenger^42^. In the pre-incubation assay, SH-SY5Y cells were incubated with 0.0001–0.1 µM **3e** for 24 h, following which R/O mixture was added and cells were incubated again. Trolox was used as a reference compound. Cell viability was 46.77% when incubated with R/O mixture alone. Cells incubated with **3e** demonstrated higher viability than with Trolox at the same concentrations (Table 3). It appears that **3e** exerts its neuroprotective activity through the activation of endogenous antioxidant pathways and is a stronger neuroprotective compound than Trolox. One-way ANOVA analysis showed, that results of **3e** and comparison with R/O treated control were not statistically significant (*p* ≤ 0.05).

In the co-incubation assay, SH-SY5Y cells were incubated with 0.1–0.0001 µM **3e**. Compound **3e** was incubated simultaneously with R/O mixture for 24 h. Cells exposed to R/O mixture alone demonstrated 47.91% viability. Cells incubated with **3e** at the concentrations of 0.001 µM, 0.0001 µM had higher viability than those incubated with Trolox at the same concentrations (Table 3). In the one-way ANOVA analysis, results were considered as not statistically significant (*p* ≤ 0.05)^41^. It can be concluded that compound **3e** demonstrated higher neuroprotective activity than Trolox and could be considered a free-radical scavenger. Neuroprotection activity was obtained at a concentration of 0.1 µM, a value similar to the IC_50_ of the AChE inhibition assay (0.131 µM).

#### In vitro inhibition studies on HYAL

The inhibitory activity of novel **3e** towards hyaluronidase was evaluated by spectrophotometric assay according to Michel et al.^45^ Compound **3e** presented low inhibitory activity towards hyaluronidase (IC_50_ 793.85 ± 1.2 µM), as indicated by comparison with heparin (IC_50_ 56.41 ± 0.78 µM) a hyaluronidase inhibitor. The compound **3e** displayed poor anti-hyaluronidase activity, which is associated with low anti-inflammatory properties.

#### Cytotoxicity – determination of hepatoprotection

The potential hepatoprotective activity of compound **3e** was determined by the MTT cytotoxicity test. The cytotoxicity of the novel compound **3e** was compared with the THA results. The range of concentration was chosen on the basis of IC_50_ results from the AChE inhibition assay. At a concentration of 10 µM, lower hepatotoxicity (higher hepatoprotection activity) was shown for **3e** (97.28% ± 4.18) than for THA (90.35% ± 3.75). This result was statistically significant (*p* < 0.1; one-way ANOVA). Both compounds (**3e** and THA) demonstrated similar viability at concentrations 1 µM and 0.1 µM, (100.40% ± 2.24, 101.52% ± 0.46 and 99.48% ± 0.74, 100.67% ± 1.03, respectively); no significant different was observed between **3e** and THA (*p* ≤ 0.1).

#### In vivo acute oral toxicity

**Figure 2.**
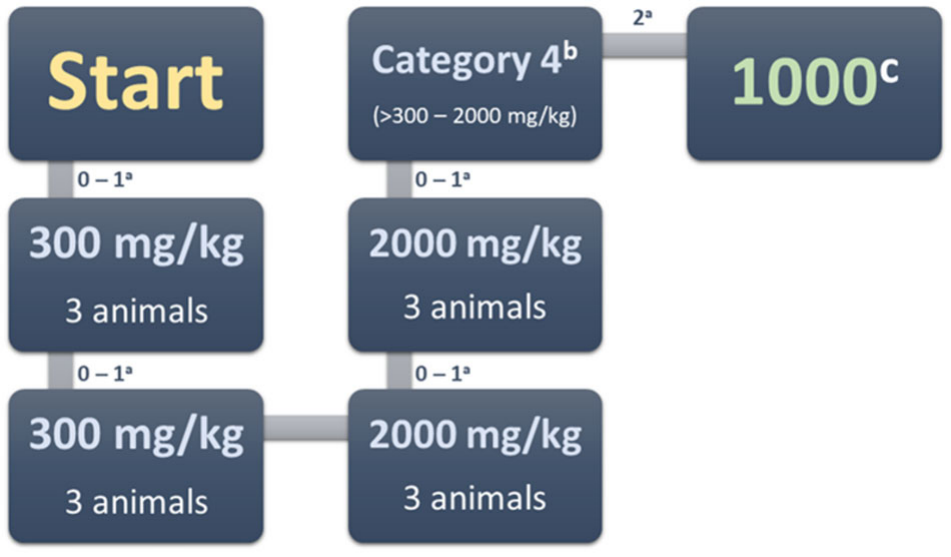
Acute oral toxicity stages. ^a^ – Moribund or dead; ^b^ – GHS (Globally Harmonised Classification System mg/kg body weight); ^c^ – LD_50_ cut-off mg/kg body weight.

Compound **3e** did not cause any death when administered at a dose of 300 mg/kg in the first and second stages (Figure 2). Therefore, a dose of 2000 mg/kg was administrated. While no animal died in stage one, two died at stage two. The compound can therefore be classified as Category 4 GHS, with an LD_50_ cut-off of 1000 mg/kg. For the sake of comparison, **3e** is less toxic than tacrine after oral administration (LD50 39.8 mg/kg)^59^. Future studies will examine the effects of intraperitoneal administration.

#### LD_50_ and toxicity class prediction

ProTox-II, a webserver for the prediction of small molecule toxicity was used to verify the LD_50_ and toxicity class of the new compounds before in vivo assay^60^. The results are expressed in Table 4. It was predicted that the new synthesised compounds would have an LD_50_ five-fold higher than tacrine and similar to bis-7-tacrine. Compound **2a** with two carbons in the alkyl chain demonstrated an LD_50_ 52.5-times higher than tacrine and 5.25-times higher than bis-7-tacrine has the same LD_50_ to bis-7-tacrine. All compounds were predicted a higher toxicity class (3–5) than tacrine.

**Table 4.** LD50 and toxicity class prediction of tacrine, bis-7-tacrine and new hybrids with 3,5-dichlorocarboxylic acid.

Compound	Predicted LD50 (mg/kg)	Predicted toxicity class
**2a**	2100	5
**2b**	200	3
**2c**	400	4
**2d**	400	4
**2e**	400	4
**2f**	400	4
**2g**	400	4
**2h**	400	4
Tacrine	40	2
Bis-7-tacrine	400	4

The prediction was performed with ProTox-II (http://tox.charite.de/protox_II), a webserver for the prediction of small molecules toxicity^53^.

#### Y3H assay

Our results confirm the presence of an interaction between ACHE and BACE-1 mediated by **3e** in *in vivo* conditions. These findings are consistent with qualitative data concerning the interaction of cyclopentaquinoline derivative with ACHE and BACE-1A^39^. The interaction was found to be of moderate strength, possibly due to the structure of the ACHE and BACE-1 active sites and the affinity of the hybrid ligand to both enzymes. Previous research suggests that the activation of ẞ-galactosidase transcription correlates with the ligand binding, although the dynamic range is only one order of magnitude^61^. The results of initial positive and negative controls are consistent with data suggested in the Matchmaker Gold Y2H system manual. In addition, the bait (ACHE) did not demonstrate any auto-activation properties; hence, the experiment could be continued. The potential protein-protein interaction between the bait (ACHE) and any of the preys (A4, BACE-1A, MAO B, MAOPT) was analysed by Y2H screen. It is possible that such protein-protein interactions could interfere with the Y3H experiment. No blue colonies were observed on DDO/X/A agar plates following the small-scale mating process. Negative results, and hence a lack of protein-protein interactions, were observed for all four combinations of bait and prey.

The present test was performed using combinations of bait and preys in the presence of **3e** that could induce protein interactions. Of all tested combinations, only the ACHE-BACE-1A protein pair yielded blue colonies, on the DDO plates, thus indicating possible interactions. Positive interactions remained stable on the more stringent QDO/X/A agar medium supplemented with 10 µM of **3e** but diminished after **3e** was removed. The findings suggest that the three tested hybrid-ligands induced interactions only between ACHE and BACE-1A.

Quantitative ẞ-galactosidase assay found the strength of the interaction mediated by **3e** to be moderate (206.37 ± 4.85 units). The dexamethasone-trimethoprim hybrid ligand previously demonstrated significantly higher ẞ-galactosidase activities (about 500 units)^62^, while the methotrexate-SLF hybrid ligand demonstrated lower activity in another Y3H system (about 150 units)^61^.

#### β-Amyloid assays

The inhibition of self-induced Aβ(1–42) aggregation was measured using a thioflavin T (ThT)-binding assay. Inhibition activities are listed in Table 5 as inhibition ratios at five test concentrations (5, 10, 25, 50 and 100 µM). Compound **3e** gave good results, with inhibition ratios from 55.7% (at 5 µM) to 82.6% (at 100 µM).

**Table 5.** Inhibition of β-amyloid (Aβ) aggregation by compound **3e** at different concentrations. Thioflavin T assay (*λ*_exc_ = 446 nm; *λ*_em_ = 490 nm).

Concentration of **3e** compound (µM)	Inhibition of Aβ(1-42) aggregation (%)
5	55.7 ± 4.7
10	62.7 ± 5.3
25	76.5 ± 3.7
50	79.8 ± 2.9
100	82.6 ± 1.7

In close concordance with Aβ42 *in vitro* assays, **3e** compound displays an anti-amyloid capacity of 52.4 with a SEM of 4.2 at 10 µM in *in cellulo* assays performed in *E. coli* system. The obtained data suggests that **3e** compound effectively cross the bacterial membrane and is fully available to inhibit Aβ42 aggregation also in this in-vivo system.

#### LogP and pKa assay

**Figure 3.**
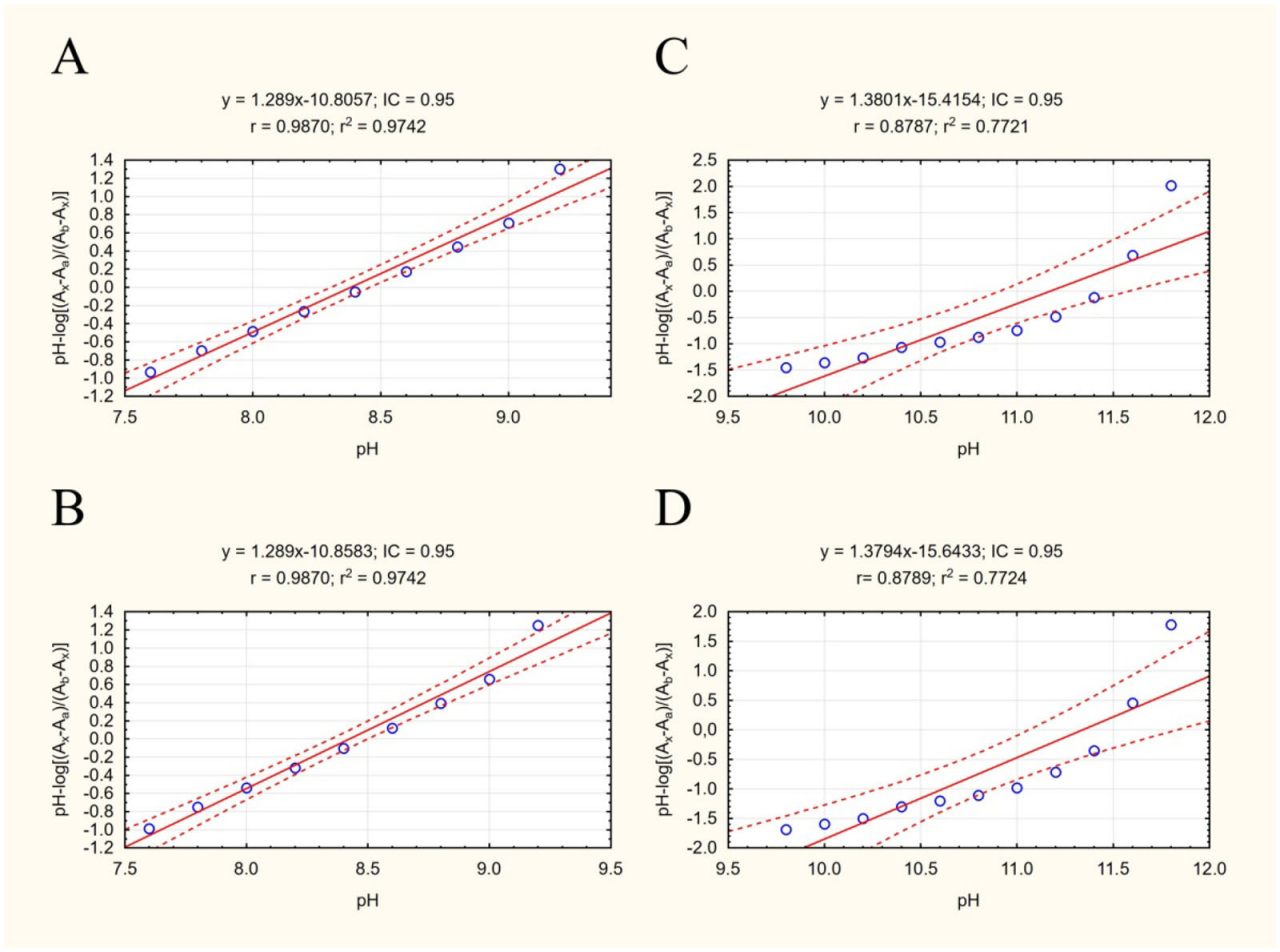
Scatterplots of equation results including a regression line, marked as the continuous line. The dashed lines determine the area of the regression belt at a confidence level of 0.95. (A) Plot pH vs pH-log(Ax-Aa)/(Ab-Ax) 332/343 nm; (B**)** plot pH vs pH-log(Ax-Aa)/(Ab-Ax) 343/332 nm; (C) plot pH vs pH-log(Ax-Aa)/(Ab-Ax) 332/343 nm; (D) plot pH vs pH-log(Ax-Aa)/(Ab-Ax) 343/332 nm.

For a drug to be used in AD therapy, it must be able to penetrate the blood brain barrier (BBB); its ability to do so is indicated by its LogP value. However, as the LogP assay requires uncharged molecules for analysis, and our studied cyclopentaquinoline derivatives change ionisation state according to pH, the present study also included a pKa assay to obtain the most reliable results. The procedure was performed as described previously^39^ according to Musil et al.^63^ Both pKa_1_ and pKa_2_ were estimated by measuring absorbance ratios at specific pH values for each ionised form (Figure 3). Our pKa_1_ values are slightly lower than those calculated by ChemAxon software and ACD/Percepta, and the experimental pKa_2_ value is much lower. All results are collected in Table 6.

**Table 6.** Experimental and predicted pKa and logP values of **3e**.

	Experimental	ChemAxon	ACD/Percepta
pKa_1_	8.40	8.89	9.17
pKa_2_	11.26	14.16	13.86
logP	5.438	5.80	6.04

**Figure 4.**
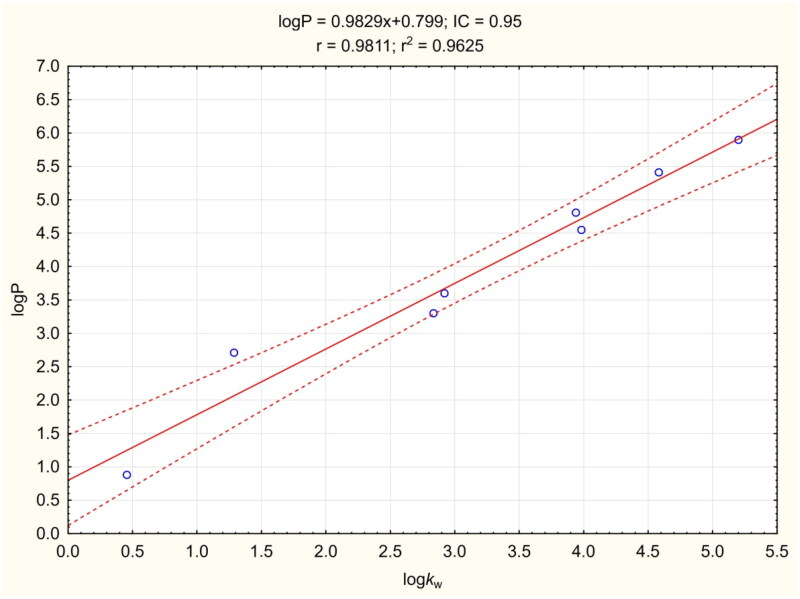
Calibration curve for LogP assay. The regression line is marked as a continuous line. Dashed lines determine the area of the regression belt at a confidence level of 0.95.

The logP procedure was performed according to Chao Liang^57^ with modifications, and allowed fast, simple and cheap determination of logP. Knowing the experimental pKa values, TEA was used as a buffering factor in the mobile phases. The coefficient of determination for the calibration curve was greater than 0.96 (Figure 4). The LogP value of compound **3e** was found to be 5.438, which was lower than the predicted values (Table 6). This result indicates that **3e** should have better BBB penetration properties than predicted computationally.

#### ADMET analysis

ADMET prediction was carried out based on the experimental values of logP and pKa1 from the previous test. The prediction was performed using ACD/Percepta software version 14.0.0 (Advanced Chemistry Development, Inc., Metropolitan Toronto, Canada). The findings confirm that **3e** demonstrates good BBB penetration to allow central nervous system (CNS) activity (logPS = −1.6). The compound is able to penetrate to the brain tissue: logBB was 0.30, with fraction unbound in plasma 0.015 and fraction unbound in brain 0.01. The compound has potentially low genotoxicity: the probability of a positive Ames test was 0.32. In addition, **3e** is close to fulfilling Lipinsky’s “Rule of Five”: it has a smaller molecular weight than 500, the correct amount of hydrogen donors and acceptors, and a topological polar surface area (TPSA) value lower than 140. The compound also has a good CNS activity profile, despite having an experimental logP higher than 5, indicating that **3e** has a good profile as a potential AD drug.

#### Docking studies and molecular dynamics simulation

**Figure 5.**
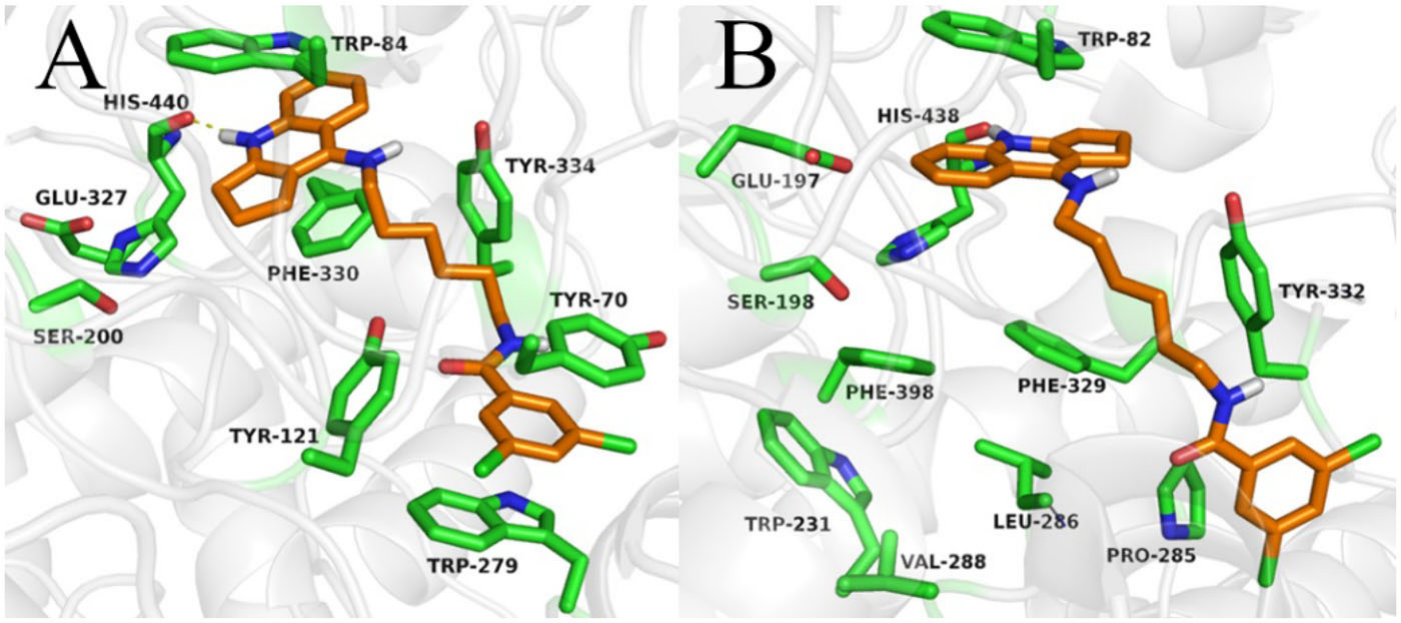
Binding mode of compound **2e** within acetylcholinesterase (A) and butyrylcholinesterase (B).

The synthesised compounds were docked to acetyl- and butyrylcholinesterase to explain their binding modes. For all compounds docked to AChE, the cyclopentaquinoline fragment was located almost identically to the tacrine from bis-(7)-tacrine in the 2CKM crystal structure. This arrangement created a characteristic sandwich due to π-π stacking and cation-π interactions with Trp84 and Phe330 in the anionic site. A hydrogen bond was observed between the protonated nitrogen atom and carbonyl group of the main chain of His440. Compounds with a short carbon linker (**2a**, **2 b**, **2c**) occurred in a bent conformation, in which the dichlorobenzamide moiety created numerous hydrophobic interactions with Tyr334, Tyr121, Tyr70 and Trp279 in the peripheral anionic site and with Phe331 in the anionic site. The amide nitrogen atom of compound **2c** also created hydrogen bound with the hydroxyl group of Tyr121. As well as the bent conformation, compound **2d** also demonstrated beneficial extended conformations, in which the dichlorophenyl ring created π–π stacking with Trp279 and Tyr70 in the peripheral anionic site. Compound **2e** demonstrated the highest activity towards AChE and BuChE; this may be due to the fact that it presented consistent, high-rated poses in an extended conformation, thus facilitating beneficial interactions (Figure 5). Compounds **2f**, **2g** and **2h** also occurred in extended conformation. Their long carbon linkers formed hydrophobic interactions with Tyr334 and Tyr121.

The binding mode of compound **2e** was confirmed by molecular dynamics simulation. The position of inhibitor was stable, and the main interactions were observed in the whole course of MD simulation. Both cyclopentaquinoline and benzamide scaffolds kept “sandwich-like” π–π interactions, while the linker represented the most flexible part of compound molecule, adopting a few various conformations (supplementary materials).

While docking to BuChE, as in case of AChE, the fragment of the tacrine analogue with a cyclopentane ring was located in the anionic site, creating π-π stacking and cation-π interactions with Trp82, as well as a hydrogen bond with the carbonyl group of the main chain of His438. The arrangement of the dichlorobenzamide fragments was very varied. Compounds **2a** and **2b** occurred in a bent conformation, creating a CH-π interaction with Trp231 and numerous hydrophobic interactions with Phe329 and Phe398, as well as with Val288 and leu286 in the acyl pocket. Compound **2c** occurred in an extended conformation, in which the dichlorophenyl substituent was oriented towards the enzyme entrance. This arrangement allowed the formation of a hydrogen bond between the amide nitrogen atom and the carbonyl group of the main chain of Pro285. Compound **2d** also presented an extended conformation; however, it did not appear to form a hydrogen bond or other beneficial interactions, accounting for its lower activity. The most active compound (**2e**) displayed an extended conformation, in which the dichlorophenyl ring formed hydrophobic interactions with Tyr332 and Pro285 (Figure 5). In case of compounds with long carbon linkers (**2f**, **2g**, **2h**), the dichlorophenyl ring formed hydrophobic interactions with Tyr282 on the enzyme surface, as well as Pro285 and Ile356. Carbon linkers were located along the enzyme cavity and interacted mainly with Tyr332.

## Conclusions

The present study describes the design and synthesis of a series of cyclopentaquinoline derivatives and their evaluation as multitarget-directed anti-Alzheimer agents. In summary, oxidative stress is one of the major contributors to AD. For this reason, compound **3e**, which was the best acetylcholinesterase inhibitor from all new cyclopentaquinoline derivatives, has been extensively tested for inhibition of oxidative stress. At most of the concentrations used in studies **3e** compound increased cell viability which is equivalent to inhibiting oxidative stress. It examined their inhibitory activities towards ChEs and Aβ(1–42) aggregation. The majority of new compounds and previously published tetrahydroacridine derivatives with dichlorobenzoic acid were found to effectively inhibit acetyl and butyrylcholinesterase in the submicromolar range. Interestingly, the most active derivative with 6 carbon atoms in the chain (**3e**) for cyclopentaquinoline derivatives in the tetrahydroacridine hybrids did not show inhibition’s activity (where the most active was a compound with 4 carbon atoms in the aliphatic chain). In the series of tetrahydroacridine derivatives there were 2 compounds that showed IC_50_ above 10 µM, while among the cyclopentaquinoline derivatives the lowest activity was about 1.5 µM, which gives the advantage of the new series. Molecular modelling studies revealed that the compounds could bind to the CAS and the PAS of enzymes. The development of dual-binding site acetylcholinesterase inhibitors is a promising approach in the search for new AD agents. Compound **3e** could inhibit both ChEs; it demonstrated the best properties in Ellman’s assay out of the tested compounds. It was also found to be non-toxic towards SHSY5Y cell at 0.1–10 µM. The results of the LogP and pKa assay indicate that it can permeate BBB, which is necessary for any substance in AD therapy. It also has good profile as a potential AD drug, indicated by ADMET prediction, and it promotes interactions between ACHE and BACE-1A, as noted by Y3H testing. **3e** did not induce DNA damage at any tested concentration. Finally, **3e** was classified as Category 4 GHS, with an LD50 cut-off of 1000 mg/kg in in vivo assay. Our findings provide useful information for further development of cyclopentaquinoline derivatives as promising lead compounds in research as potential anti-AD drug candidates.

## Supplementary Material

Supplemental Material
