# Supplementary material for: New cyclopentaquinoline and 3,5-dichlorobenzoic acid hybrids with neuroprotection against oxidative stress for the treatment of Alzheimer’s disease
Source: J Enzyme Inhib Med Chem. 2023 Jan 11;38(1):2158822. doi: 10.1080/14756366.2022.2158822 (PMC9848259; doi:10.1080/14756366.2022.2158822)

Supplementary materials

New cyclopentaquinoline and 3,5-dichlorobenzoic acid hybrids with neuroprotection against oxidative stress for the treatment of Alzheimer's disease

Kamila Czarnecka[1]*, Małgorzata Girek[1], Paweł Kręcisz[1], Robert Skibiński[2], Kamil Łątka[3], Jakub Jończyk[3], Marek Bajda[3], Piotr Szymczyk[4], Grzegorz Galita[5], Jacek Kabziński[5], Ireneusz Majsterek[5], Alba Espargaró[6,7], Raimon Sabate[6,7], Paweł Szymański[1,8]*

[1] *Department of Pharmaceutical Chemistry, Drug Analyses and Radiopharmacy, Faculty of Pharmacy, Medical University of Lodz, Muszyńskiego 1, 90-151 Lodz, Poland;*

[2] *Department of Medicinal Chemistry, Faculty of Pharmacy, Medical University of Lublin, Jaczewskiego 4, 20-090 Lublin, Poland;*

[3] *Department of Physicochemical Drug Analysis, Chair of Pharmaceutical Chemistry, Faculty of Pharmacy, Jagiellonian University Medical College, Medyczna 9, 30-688 Krakow, Poland;*

[4] *Department of Pharmaceutical Biotechnology, Faculty of Pharmacy, Medical University of Lodz, Muszyńskiego 1, 90-151 Lodz, Poland;*

[5] *Department of Clinical Chemistry and Biochemistry, Medical University of Lodz, Narutowicza 60, 90-647 Lodz, Poland;*

[6] *Department of Pharmacy and Pharmaceutical Technology and Physical Chemistry, Faculty of Pharmacy and Food Sciences, University of Barcelona, Av. Joan XXIII, 27-31, E-08028 Barcelona, Spain;*

[7] *Institute of Nanoscience and Nanotechnology (IN2UB), 645 Diagolan Ave, 08028 Barcelona, Spain*

[8] *Department of Radiobiology and Radiation Protection, Military Institute of Hygiene and Epidemiology, Kozielska 4, 01-001 Warsaw, Poland*

* Correspondence: K.C., Department of Pharmaceutical Chemistry, Drug Analyses and Radiopharmacy, Faculty of Pharmacy, Medical University of Lodz, Muszyńskiego 1, 90-151 Lodz, Poland Email:kamila.czarnecka@umed.lodz.pl; P.S., Department of Pharmaceutical Chemistry, Drug Analyses and Radiopharmacy, Faculty of Pharmacy, Medical University of Lodz, Muszyńskiego 1, 90-151 Lodz, Poland Email: pawel.szymanski@umed.lodz.pl

Details about synthesis procedures of compounds 2a-2h

3,5-Dichloro-N-[2-(2,3-dihydro-1H-cyclopenta[b]quinolin-9-ylamino)-ethyl]-benzamide (2a)

Intermediate **1a** (2,3-dihydro-1H-cyclopenta[b]quinolin-9-amine derivative) and 3,5-dichloronicotinic acid were reacted according general procedure to give desired product **2a** as a cream oil (64% yield); IR (KBr) v_{max}/cm^{-1}: 1661.7, 2940.3, 3027.7, 3259.5; ^{1}H NMR (600 MHz, Methanol-d_4) δ 8.33 (1H, d, J = 9.1 Hz, Ar), 7.87 (1H, t, J = 8.3 Hz, Ar), 7.76 (1H, d, J = 9.1 Hz, Ar), 7.68 – 7.69 (2H, m, Ar), 7.65 – 7.67 (2H, m, Ar), 4.08 (2H, t, J = 5.9 Hz, CH_2), 3.76 (2H, t, J = 5.9 Hz, CH_2), 3.42 – 3.45 (2H, m, CH_2), 3.17 (2H, t, J = 7.9 Hz, CH_2), 2.30 (2H, p, J = 7.8 Hz, CH_2) (protons of NH groups invisible); MS (ESI) m/z: 94.0, 121.1, 185.1, 216.0; MS-HR (ESI) calcd for $C_{21}H_{19}Cl_2N_3O$: 399.09052, found(M+1): 400.09777.

3,5-Dichloro-N-[3-(2,3-dihydro-1H-cyclopenta[b]quinolin-9-ylamino)-propyl]-benzamide (2b)

Intermediate **1b** (2,3-dihydro-1H-cyclopenta[b]quinolin-9-amine derivative) and 3,5-

dichloronicotinic acid were reacted according general procedure to give desired product **2b** as a cream oil (68% yield); IR (KBr) v_{max}/cm^{-1}: 1672.6, 2951.9, 3019.3, 3251.9; ^{1}H NMR (600 MHz, Methanol-d_4) δ 8.37 (1H, d, J = 8.1 Hz, Ar), 7.85 (1H, t, J = 8.2 Hz, Ar), 7.78 – 7.79 (2H, m, Ar), 7.74 (1H, d, J = 9.1 Hz, Ar), 7.62 – 7.67 (1H, m, Ar), 3.92 (2H, t, J = 6.8 Hz, CH_2), 3.58 (2H, t, J = 6.4 Hz, CH_2), 3.38 – 3.42 (2H, m, CH_2), 3.19 (2H, t, J = 7.9 Hz, CH_2), 2.29 (2H, p, J = 7.8 Hz, CH_2), 2.07 (2H, p, J = 6.8 Hz, CH_2) (protons of NH groups invisible); MS (ESI) m/z: 94.0, 121.1, 185.1, 230.0, 414.1; MS-HR (ESI) calcd for $C_{22}H_{21}Cl_2N_3O$: 413.10617(without HCl), found (M+1): 414.11310.

3,5-Dichloro-N-[4-(2,3-dihydro-1H-cyclopenta[b]quinolin-9-ylamino)-butyl]-benzamide (2c)

Intermediate **1c** (2,3-dihydro-1H-cyclopenta[b]quinolin-9-amine derivative) and 3,5-dichloronicotinic acid were reacted according general procedure to give desired product **2c** as a cream oil (60% yield); IR (KBr) v_{max}/cm^{-1}: 1657.7, 2928.2, 3046.0, 3284.5; ^{1}H NMR (600 MHz, Methanol-d_4) δ 8.32 (1H, d, J = 8.4 Hz, Ar), 7.85 (1H, t, J = 8.2 Hz, Ar), 7.71 – 7.75 (3H, m, Ar), 7.61 – 7.66 (2H, m, Ar), 3.87 (2H, t, J = 7.0 Hz, CH_2), 3.45 (2H, t, J = 6.6 Hz, CH_2), 3.38 – 3.42 (2H, m, CH_2), 3.17 (2H, t, J = 7.9 Hz, CH_2), 2.28 (2H, p, J = 7.8 Hz, CH_2), 1.76 – 1.89 (4H, m, CH_2) (protons of NH groups invisible); MS (ESI) m/z: 94.0, 121.1, 185.1, 239.1, 428.1; MS-HR (ESI) calcd for $C_{23}H_{23}Cl_2N_3O$: 427.12182 , found (M+1): 428.12880.

3,5-Dichloro-N-[5-(2,3-dihydro-1H-cyclopenta[b]quinolin-9-ylamino)-pentyl]-benzamide (2d)

Intermediate **1d** (2,3-dihydro-1H-cyclopenta[b]quinolin-9-amine derivative) and 3,5-

dichloronicotinic acid were reacted according general procedure to give desired product **2d** as a cream oil (60% yield); FTIR-ATR v_{max}/cm^{-1}: 752.9, 1363.8, 1447.9, 1560.6, 1675.9, 2930.1, 3299.8; ^{1}H NMR (600 MHz, Methanol-d_4) δ 8.30 (1H, d, J = 8.1 Hz, Ar), 7.84 (1H, t, J = 8.3 Hz, Ar), 7.72 (1H, d, J = 8.4 Hz, Ar), 7.69 – 7.70 (2H, m, Ar), 7.59 – 7.63 (2H, m, Ar), 3.85 (2H, t, J = 7.1 Hz, CH_2), 3.39 – 3.45 (4H, m, CH_2), 3.19 (2H, t, J = 7.9 Hz, CH_2), 2.31 (2H, p, J = 7.8 Hz, CH_2), 1.84 (2H, p, J = 7.4 Hz, CH_2), 1.73 (2H, p, J = 7.0 Hz, CH_2), 1.55 (2H, p, J = 7.4, 7.0 Hz, CH_2). (protons of NH groups invisible); MS (ESI) m/z: 185.1, 253.1, 444.1; MS-HR (ESI) calcd for $C_{24}H_{25}Cl_2N_3O$: 441.13747, found: 441.13783.

3,5-Dichloro-N-[6-(2,3-dihydro-1H-cyclopenta[b]quinolin-9-ylamino)-hexyl]-benzamide (2e)

Intermediate **1e** (2,3-dihydro-1H-cyclopenta[b]quinolin-9-amine derivative) and 3,5-dichloronicotinic acid were reacted according general procedure to give desired product **2e** as a cream oil (70% yield); FTIR-ATR v_{max}/cm^{-1}: 761.7, 1361.1, 1469.0, 1561.9, 1632.7, 2933.9, 3219.9; ^{1}H NMR (600 MHz, Methanol-d_4) δ 8.29 (1H, d, J = 8.2 Hz, Ar), 7.82 (1H, t, J = 8.2 Hz, Ar), 7.76 – 7.78 (2H, m, Ar), 7.74 (1H, d, J = 8.4 Hz, Ar), 7.64 (1H, s, Ar), 7.60 (1H, t, J = 8.3 Hz, Ar), 3.79 – 3.82 (2H, m, CH_2), 3.37 – 3.42 (4H, m, CH_2), 3.16 (2H, t, J = 7.9 Hz, CH_2), 2.28 (2H, p, J = 7.8 Hz, CH_2), 1.79 (2H, p, J = 7.5 Hz, CH_2), 1.68 (2H, p, J = 7.2 Hz, CH_2), 1.46 – 1.57 (4H, m, CH_2) (protons of NH groups invisible); MS (ESI) m/z: 185.1, 267.2, 458.2; MS-HR (ESI) calcd for $C_{25}H_{27}Cl_2N_3O$: 455.15312, found: 455.15312.

3,5-Dichloro-N-[7-(2,3-dihydro-1H-cyclopenta[b]quinolin-9-ylamino)-heptyl]-benzamide (2f)

Intermediate **1f** (2,3-dihydro-1H-cyclopenta[b]quinolin-9-amine derivative) and 3,5-dichloronicotinic acid were reacted according general procedure to give desired product **2f** as a cream oil (69% yield); FTIR-ATR v_{max}/cm^{-1}: 763.2, 1372.5, 1468.9, 1560.5, 1633.3, 2930.1, 3219.1; ^{1}H NMR (600 MHz, Methanol-d_4) δ 8.34 (1H, d, J = 8.3 Hz, Ar), 7.87 (1H, t, J = 8.2 Hz, Ar), 7.74 – 7.78 (3H, m, Ar), 7.61 – 7.67 (1H, m, Ar), 3.82 – 3.85 (2H, m, CH_2), 3.36 – 3.42 (4H, m, CH_2), 3.20 (2H, t, J = 7.9 Hz, CH_2), 2.32 (2H, p, J = 7.8 Hz, CH_2), 1.80 (2H, p, J = 7.7 Hz, CH_2), 1.65 (2H, p, J = 7.3 Hz, 2H), 1.42 – 1.54 (6H, m, CH_2) (protons of NH groups invisible); MS (ESI) m/z: 185.1, 281.2, 472.1; MS-HR (ESI) calcd for $C_{26}H_{29}Cl_2N_3O$: 469.16877, found: 469.16991.

3,5-Dichloro-N-[8-(2,3-dihydro-1H-cyclopenta[b]quinolin-9-ylamino)-octyl]-benzamide (2g)

Intermediate **1g** (2,3-dihydro-1H-cyclopenta[b]quinolin-9-amine derivative) and 3,5-dichloronicotinic acid wre reacted according general procedure to give desired product **2g** as a cream oil (63% yield); FTIR-ATR v_{max}/cm^{-1}: 761.1, 1356.2, 1468.5, 1560.4, 1632.5, 2926.0, 3186.4; ^{1}H NMR (600 MHz, Methanol-d_4) δ 8.21 (1H, d, J = 8.5 Hz, Ar), 7.77 – 7.79 (2H, m, Ar), 7.71 – 7.76 (2H, m, Ar), 7.63 (1H, s, Ar), 7.53 (1H, t, J = 8.3 Hz, Ar), 3.71 – 3.75 (2H, m, CH_2), 3.35 – 3.39 (4H, m, CH_2), 3.10 (2H, t, J = 7.8 Hz, CH_2), 2.24 (2H, p, J = 7.7 Hz, CH_2), 1.74 (2H, p, J = 7.4 Hz, CH_2), 1.60 – 1.65 (2H, m, CH_2), 1.38 – 1.51 (8H, m, CH_2) (protons of NH groups invisible); MS (ESI) m/z: 185.1, 295.2, 486.2; MS-HR (ESI) calcd for $C_{27}H_{31}Cl_2N_3O$: 483,18442, found: 483.18537.

3,5-Dichloro-N-[9-(2,3-dihydro-1H-cyclopenta[b]quinolin-9-ylamino)-nonyl]-benzamide (2h)

Intermediate **1h** (2,3-dihydro-1H-cyclopenta[b]quinolin-9-amine derivative) and 3,5-dichloronicotinic acid were reacted according general procedure to give desired product **2h** as a cream solid (58% yield); FTIR-ATR v_{max}/cm^{-1}: 757.3, 1360.7, 1456.2, 1564.5, 1645.5, 2924.3, 3244.7; ^{1}H NMR (600 MHz, Methanol-d_4) δ 8.26 (1H, d, J = 8.5 Hz, Ar), 7.73 – 7.81 (4H, m, Ar), 7.63 (1H, s, Ar), 7.58 (1H, t, J = 8.3 Hz, Ar), 3.74 – 3.78 (2H, m, CH_2), 3.34 – 3.38 (4H, m, CH_2), 3.14 (2H, t, J = 7.8 Hz, CH_2), 2.27 (2H, p, J = 7.7 Hz, CH_2), 1.75 (2H, p, J = 7.5 Hz, CH_2), 1.62 (2H, p, J = 7.3 Hz, CH_2), 1.35 – 1.51 (10H, m, CH_2) (protons of NH groups invisible); MS (ESI) m/z: 185.1, 309.2, 500.2; MS-HR (ESI) calcd for $C_{28}H_{33}Cl_2N_3O$: 497.20007, found: 497.19999.

Details about synthesis procedures of compounds 3a-3h

3,5-Dichloro-N-[2-(2,3-dihydro-1H-cyclopenta[b]quinolin-9-ylamino)-ethyl]-benzamide hydrochloride (3a)

Compound 3a: Yield: 44%; white solid; mp 261-263 ºC; IR (KBr) v_{max}/cm^{-1}:): 1635.2, 2952.5, 3037.9, 3260.0, 3433.0; ^{1}H NMR (500 MHz, DMSO-d_6) δ 14.08 (1H, s, HCl), 9.05 (1H, t, *J* = 5.7 Hz, Ar), 8.45 (1H, d, *J* = 8.6 Hz, Ar), 7.73 – 7.85 (4H, m, Ar), 7.60 (1H, t, *J* = 8.3 Hz, Ar), 3.87 (2H, q, *J* = 6.2 Hz, CH_2), 3.54 (2H, q, *J* = 6.1 Hz, CH_2), 3.27 (2H, t, *J* = 7.3 Hz, CH_2), 3.07 (2H, t, *J* = 7.9 Hz, CH_2), 2.11 (2H, p, *J* = 7.7 Hz, CH_2) (protons of NH groups invisible); MS (ESI) m/z: 94.0, 121.1, 185.1, 216.0, 400.1; MS-HR (ESI) calcd for $C_{21}H_{19}Cl_2N_3O$: 399.09052 (without HCl); found (M+1): 400.09757

3,5-Dichloro-N-[3-(2,3-dihydro-1H-cyclopenta[b]quinolin-9-ylamino)-propyl]-benzamide hydrochloride (3b)

Compound 3b: Yield: 48%; white solid; mp 256-258 ºC; IR (KBr) v_{max}/cm^{-1}: 1671.1, 2936.0, 3043.2, 3256.7, 3404.1; ^{1}H NMR (500 MHz, DMSO-d_6) δ 13.89 (1H, s, HCl), 8.86 (1H, t, *J* = 5.6 Hz, Ar), 8.42 (1H, d, *J* = 8.5 Hz, Ar), 7.72 – 7.84 (4H, m, Ar), 7.58 (1H, t, *J* = 7.8 Hz, Ar), 3.75 (2H, q, *J* = 6.7 Hz, CH_2), 3.45 – 3.43 (2H, m, CH_2), 3.23 (2H, t, *J* = 7.2 Hz, CH_2), 3.07 (2H, t, *J* = 7.9 Hz, CH_2), 2.08 (2H, p, *J* = 7.7 Hz, CH_2), 1.90 (2H, p, *J* = 6.7 Hz, CH_2), (protons of NH groups invisible); MS (ESI) m/z: 94.0, 121.1, 185.1, 230.0, 414.1; MS-HR (ESI): calcd. for $C_{22}H_{21}Cl_2N_3O$: 413.10617 (without HCl); found (M+1): 414.11322

3,5-Dichloro-N-[4-(2,3-dihydro-1H-cyclopenta[b]quinolin-9-ylamino)-butyl]-benzamide hydrochloride (3c)

Compound 3c: Yield: 58%; white solid; mp 139-141 ºC; IR (KBr) v_{max}/cm^{-1}: 1656.9, 2925.3, 3047.4, 3280.9, 3415.4; ^{1}H NMR (500 MHz, DMSO-d_6) δ 13.95 (1H, s, HCl), 8.72 (1H, t, J = 5.5 Hz, Ar), 8.42 (1H, d, J = 8.5 Hz, Ar), 7.73 – 7.83 (4H, m, Ar), 7.57 (1H, d, J = 8.2 Hz, Ar), 3.69 (2H, q, J = 6.7 Hz, CH_2), 3.21 – 3.30 (4H, m, CH_2), 3.07 (2H, t, J = 7.9 Hz, CH_2), 2.09 (2H, p, J = 7.8 Hz, CH_2), 1.57 – 1.71 (4H, m, CH_2), (protons of NH groups invisible); MS (ESI) m/z: 94.0, 121.1, 185.1, 239.1, 428.1; MS-HR (ESI): calcd. for $C_{23}H_{23}Cl_2N_3O$: 427.12182 (without HCl); found (M+1): 428.12878

3,5-Dichloro-N-[5-(2,3-dihydro-1H-cyclopenta[b]quinolin-9-ylamino)-pentyl]-benzamide hydrochloride (3d)

Compound 3d: Yield: 63%; white solid; mp 135-137 ºC; FTIR (ATR) v (cm^{-1}): 760.7, 1369.4, 1457.1, 1563.2, 1633.9, 2930.7, 3205.4; ^{1}H NMR (500 MHz, DMSO-d_6) δ 13.90 (1H, s, HCl), 8.65 (1H, t, J = 5.6 Hz, Ar), 8.41 (1H, d, J = 8.5 Hz, Ar), 7.73 – 7.83 (4H, m, Ar), 7.56 (1H, t, J = 8.2 Hz, Ar), 3.67 (2H, q, J = 6.9 Hz, CH_2), 3.21 – 3.26 (4H, m, CH_2), 3.08 (2H, t, J = 7.9 Hz, CH_2), 2.11 (2H, p, J = 7.7 Hz, CH_2), 1.66 (2H, p, J = 7.6 Hz, CH_2), 1.54 (2H, p, J = 7.0 Hz, CH_2), 1.36 (2H, p, J = 7.3, 6.7 Hz, CH_2) (protons of NH groups invisible); MS (ESI) m/z: 185.1, 253.2, 444.1; MS-HR (ESI): calcd. for $C_{24}H_{25}Cl_2N_3O$: 441.13747 (without HCl); found: 441.13763

3,5-Dichloro-N-[6-(2,3-dihydro-1H-cyclopenta[b]quinolin-9-ylamino)-hexyl]-benzamide hydrochloride (3e)

Compound 3e: Yield: 38%; white solid; mp 126-127 ºC; FTIR (ATR) v (cm^{-1}): 760.6,

1362.3, 1459.5, 1562.5, 1633.7, 2932.4, 3206.7; ^{1}H NMR (500 MHz, DMSO-d_6) δ 13.86 (1H, s, HCl), 8.65 (1H, t, J = 4.4 Hz, Ar), 8.41 (1H, d, J = 8.5 Hz, Ar), 7.73 – 7.84 (4H, m, Ar), 7.56 – 7.61 (1H, m, Ar), 3.66 (2H, q, J = 6.7 Hz, CH_2), 3.19 – 3.25 (4H, m, CH_2), 3.08 (2H, t, J = 7.9 Hz, CH_2), 2.11 (2H, p, J = 7.6 Hz, CH_2), 1.63 (2H, p, J = 7.5 Hz, CH_2), 1.46 – 1.53 (2H, m, CH_2), 1.28 – 1.40 (4H, m, CH_2), (protons of NH groups invisible); MS (ESI) m/z: 185.1, 267.2, 458.2; MS-HR (ESI): calcd. for $C_{25}H_{27}Cl_2N_3O$: 455.15312 (without HCl); found: 455.15410

3,5-Dichloro-N-[7-(2,3-dihydro-1H-cyclopenta[b]quinolin-9-ylamino)-heptyl]-benzamide hydrochloride (3f)

Compound 3f: Yield: 38%; white solid; mp 120-122 ºC; FTIR (ATR) ν (cm^{-1}): 758.4, 1362.0,1465.8, 1562.7, 1633.0, 2927.3, 3220.1; ^{1}H NMR (500 MHz, DMSO-d_6) δ 13.89 (1H, s, HCl), 8.65 (1H, t, J = 5.5 Hz, Ar), 8.41 (1H, d, J = 8.6 Hz, Ar), 7.73 – 7.85 (4H, m, Ar), 7.56 – 7.61 (1H, m, Ar), 3.65 (2H, q, J = 6.8 Hz, CH_2), 3.18 – 3.26 (4H, m, CH_2), 3.08 (2H, t, J = 7.8 Hz, CH_2), 2.12 (2H, p, J = 7.7 Hz, CH_2), 1.57 – 1.66 (2H, m, CH_2), 1.47 (2H, p, J = 7.2 Hz, CH_2), 1.37 – 1.24 (6H, m, CH_2), (protons of NH groups invisible); MS (ESI) m/z: 185.1, 281.2, 472.2; MS-HR (ESI): calcd. for $C_{26}H_{29}Cl_2N_3O$: 469.16877 (without HCl); found: 469.16889

3,5-Dichloro-N-[8-(2,3-dihydro-1H-cyclopenta[b]quinolin-9-ylamino)-octyl]-benzamide hydrochloride (3g)

Compound 3g: Yield: 59%; white solid; mp 115-117 ºC; FTIR (ATR) ν (cm^{-1}): 757.8, 1362.1, 1464.3, 1562.6, 1633.5, 2926.6, 3225.3; ^{1}H NMR (500 MHz, DMSO-d_6) δ 13.90 (1H, s, HCl), 8.65 (1H, t, J = 5.6 Hz, Ar), 8.42 (1H, d, J = 8.6 Hz, Ar), 7.73 – 7.85 (4H,

m, Ar), 7.59 (1H, t, J = 8.2 Hz, Ar), 3.65 (2H, q, J = 6.8 Hz, CH_2), 3.17 – 3.26 (2H, m, CH_2), 3.09 (2H, t, J = 7.9 Hz, CH_2), 2.12 (2H, p, J = 7.8 Hz, CH_2), 1.61 (2H, p, J = 7.8 Hz, CH_2), 1.47 (2H, p, J = 6.6 Hz, CH_2), 1.23 – 1.37 (8H, m, CH_2), (protons of NH groups invisible); MS (ESI) m/z: 185.1, 295.2, 486.2; MS-HR (ESI): calcd. for $C_{27}H_{31}Cl_2N_3O$: 483.18442 (without HCl); found: 483.18438

3,5-Dichloro-N-[9-(2,3-dihydro-1H-cyclopenta[b]quinolin-9-ylamino)-nonyl]-benzamide hydrochloride (3h)

Compound 3h: Yield: 40%; white solid; mp 100-102 ºC; FTIR (ATR) ν (cm^{-1}): 758.6, 1362.5, 1464.1, 1559.5, 1632.3, 2923.4, 3225.6; ^{1}H NMR (500 MHz, DMSO-d_6) δ 13.92 (1H, s, HCl), 8.65 (1H, t, J = 5.0 Hz, Ar), 8.41 – 8.43 (1H, m, Ar), 7.73 – 7.84 (4H, m, Ar), 7.59 (1H, t, J = 8.2 Hz, Ar), 3.62 – 3.68 (2H, m, CH_2), 3.16 – 3.26 (4H, m, CH_2), 3.09 (2H, t, J = 7.9 Hz, CH_2), 2.12 (2H, p, J = 7.8 Hz, CH_2), 1.57 – 1.64 (2H, m, CH_2), 1.42 – 1.50 (2H, m, CH_2), 1.20 – 1.37 (10H, m, CH_2), (protons of NH groups invisible); MS (ESI) m/z: 185.1, 309.2, 500.2; MS-HR (ESI): calcd. for $C_{28}H_{33}Cl_2N_3O$: 497.20007 (without HCl); found: 497.20012

MS spectra of compounds 2a-2h

3,5-Dichloro-N-[2-(2,3-dihydro-1H-cyclopenta[b]quinolin-9-ylamino)-ethyl]-benzamide (2a)

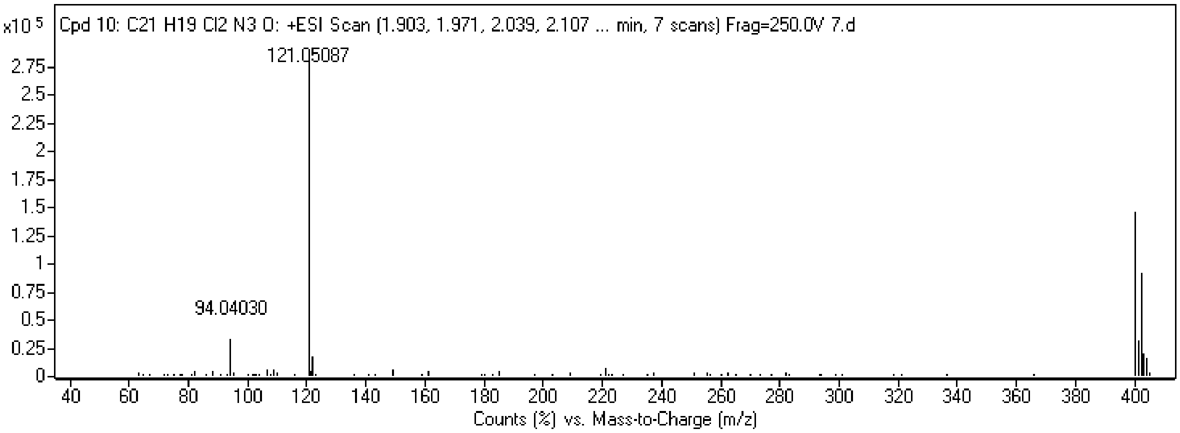

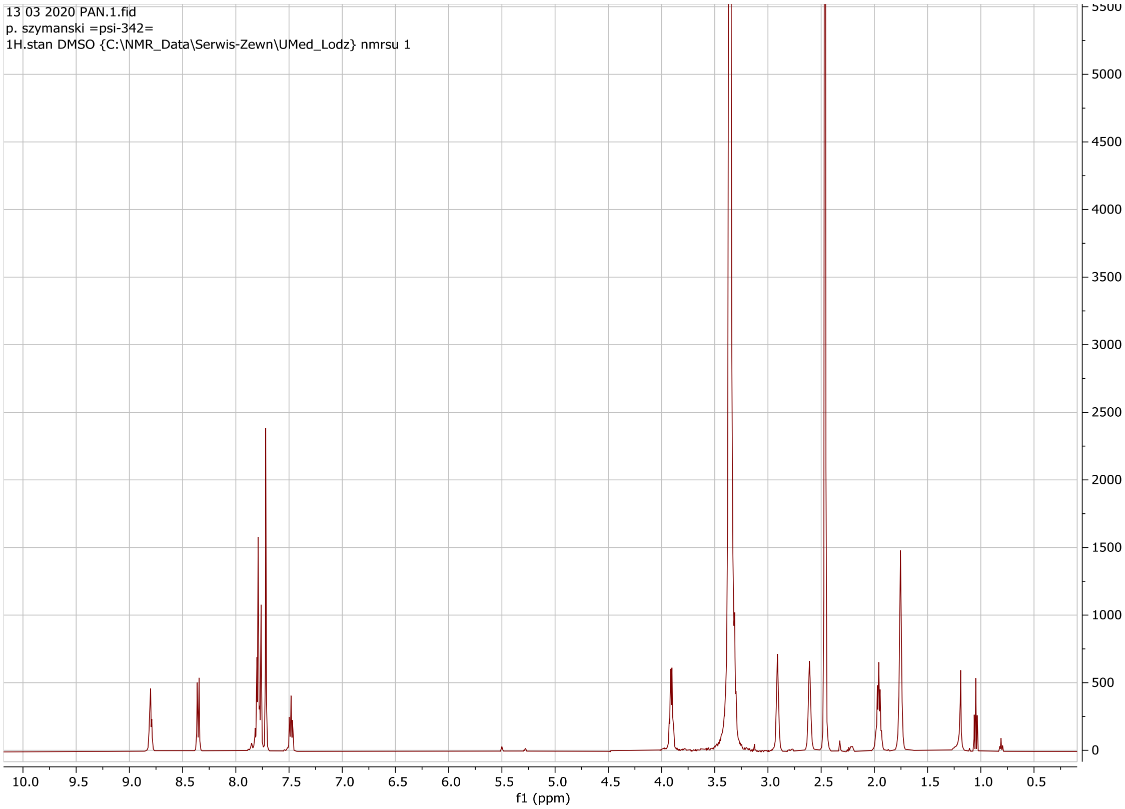

3,5-Dichloro-N-[3-(2,3-dihydro-1H-cyclopenta[b]quinolin-9-ylamino)-propyl]-benzamide (2b)

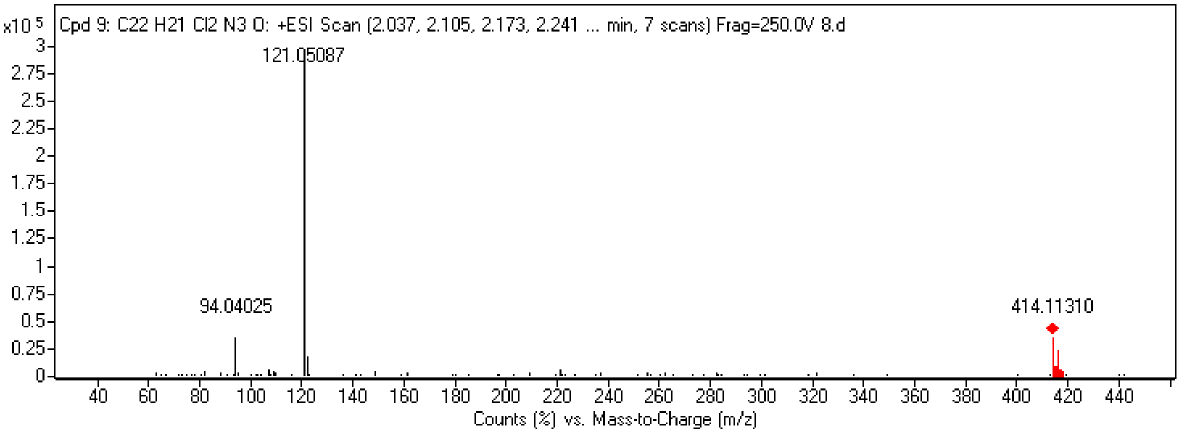

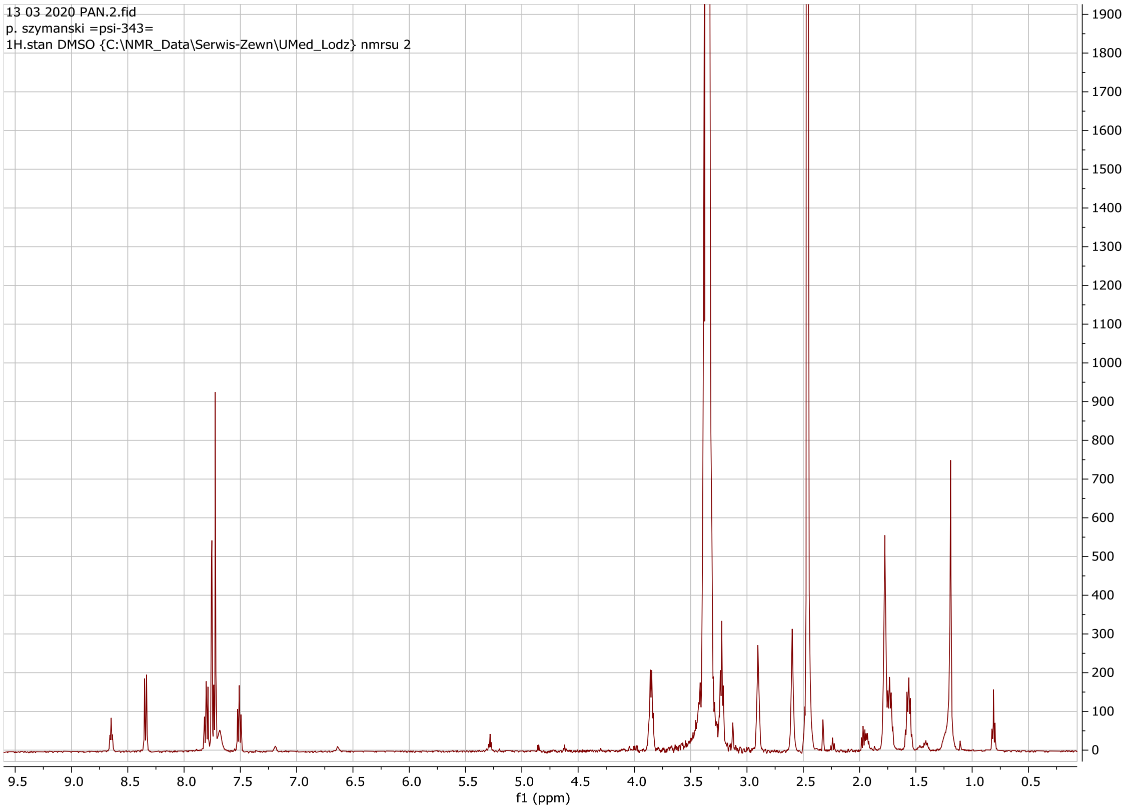

3,5-Dichloro-N-[4-(2,3-dihydro-1H-cyclopenta[b]quinolin-9-ylamino)-butyl]-benzamide (2c)

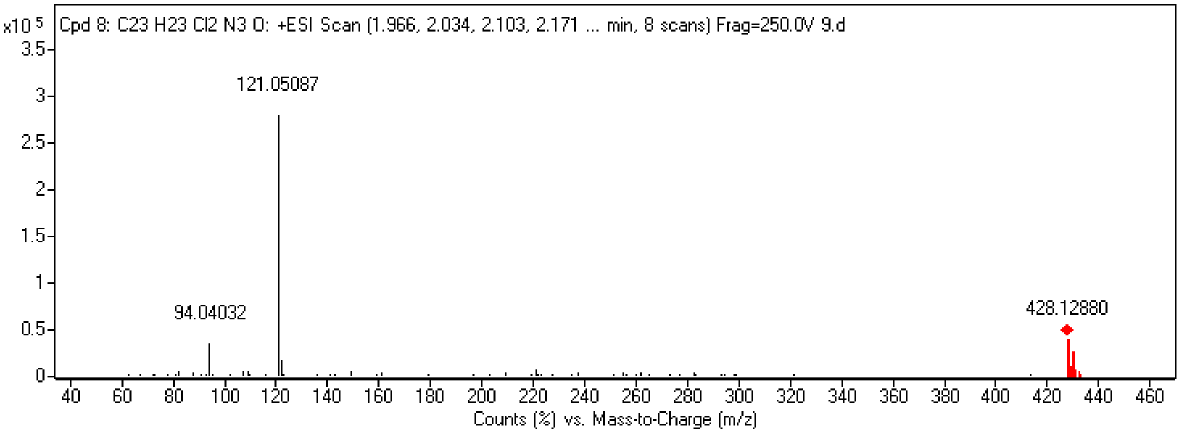

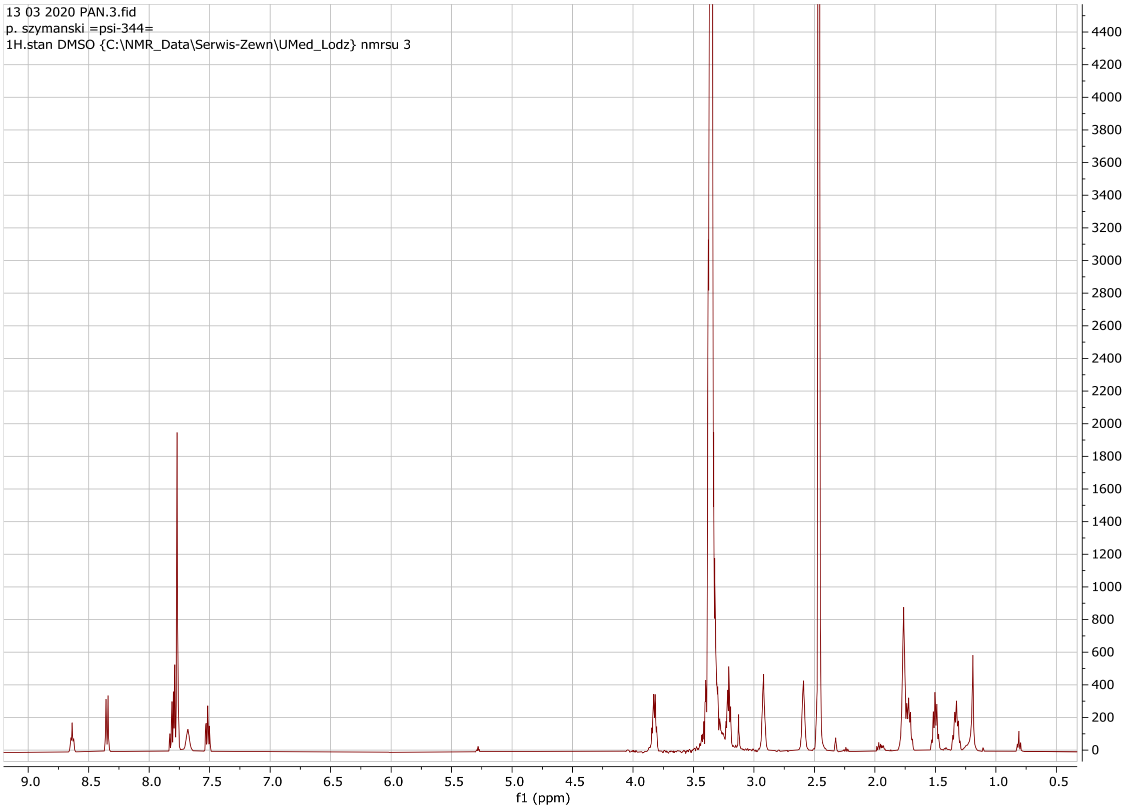

3,5-Dichloro-N-[5-(2,3-dihydro-1H-cyclopenta[b]quinolin-9-ylamino)-pentyl]-benzamide (2d)

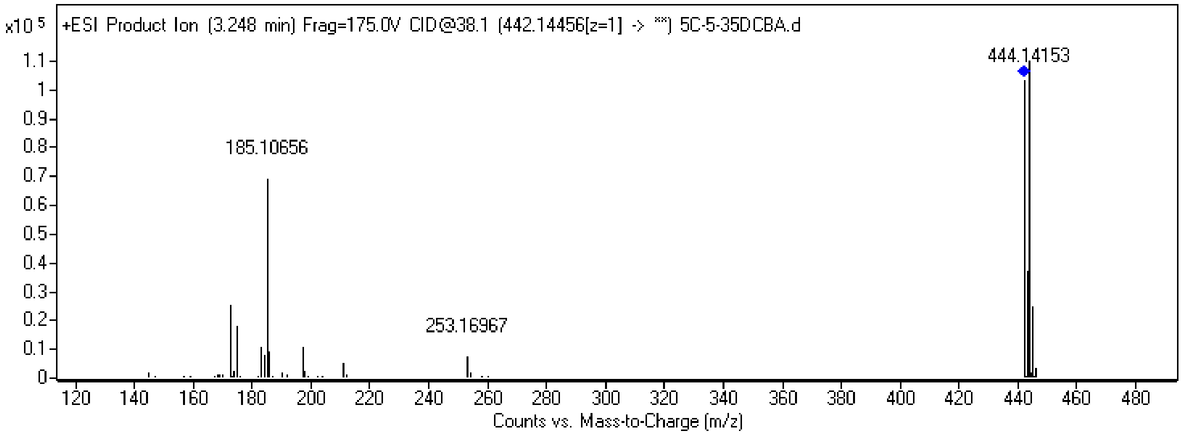

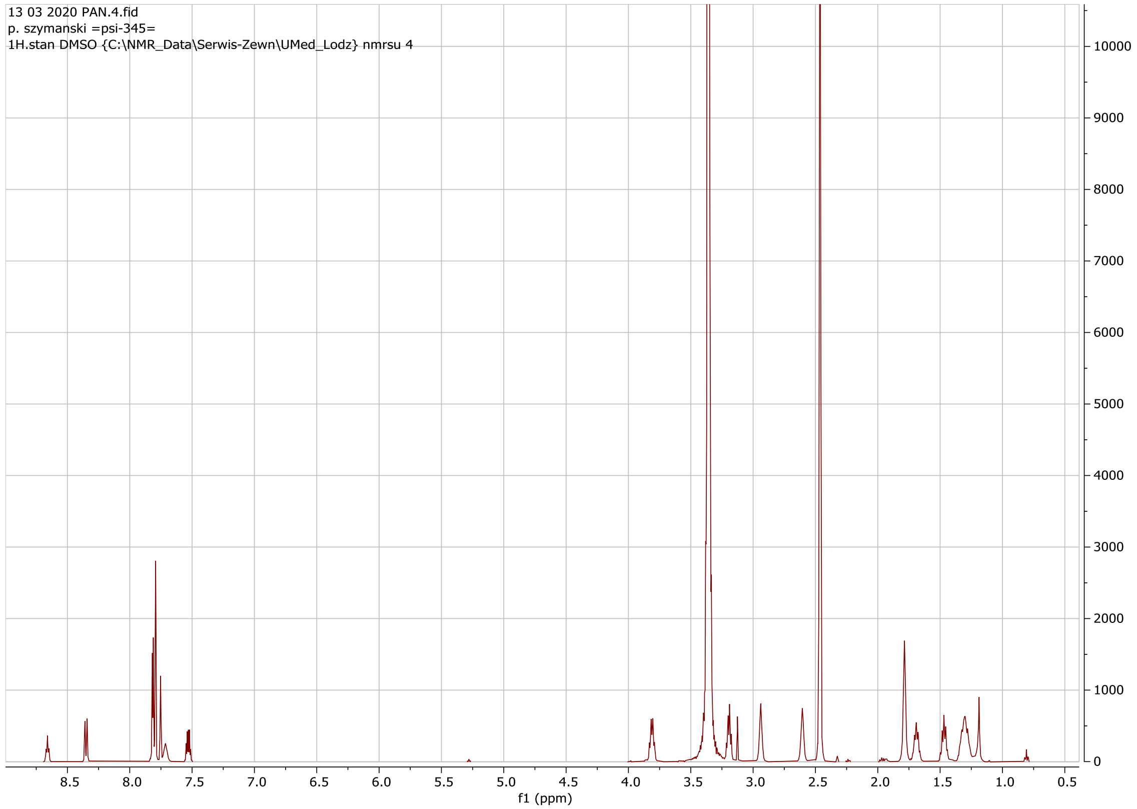

3,5-Dichloro-N-[6-(2,3-dihydro-1H-cyclopenta[b]quinolin-9-ylamino)-hexyl]-benzamide (2e)

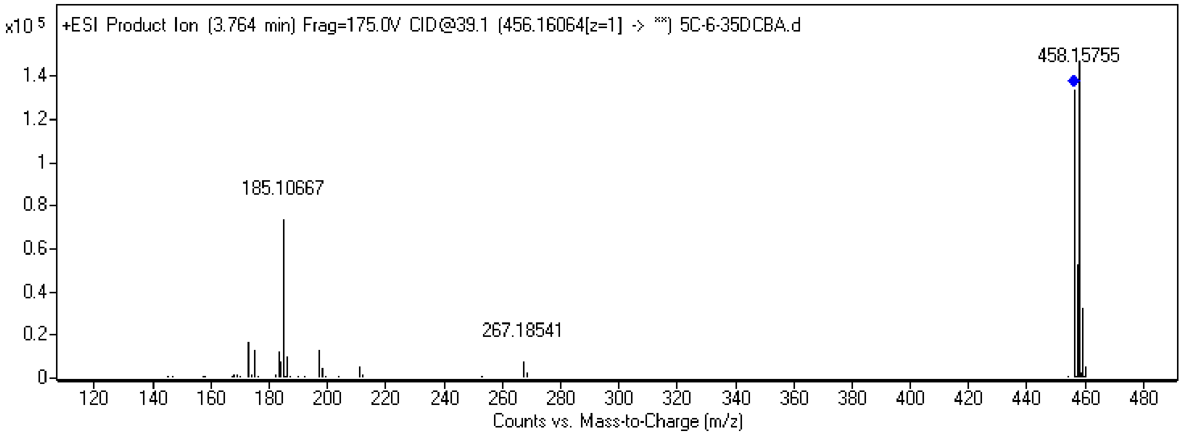

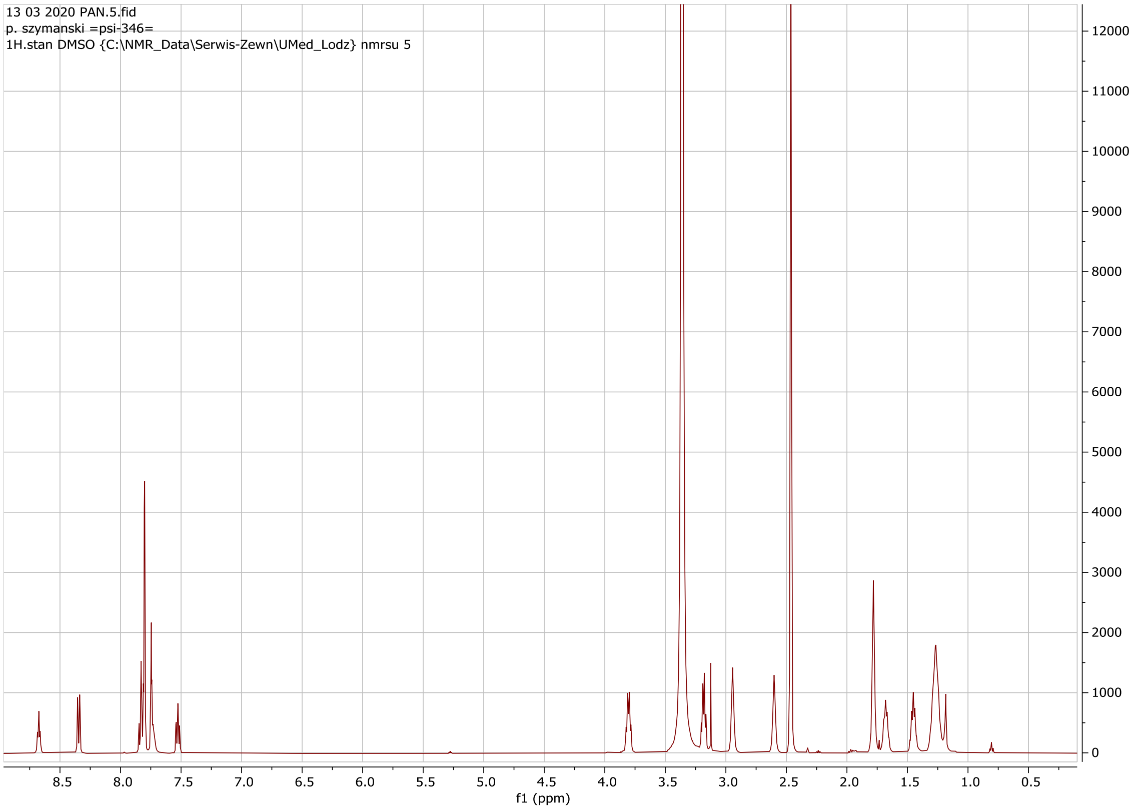

3,5-Dichloro-N-[7-(2,3-dihydro-1H-cyclopenta[b]quinolin-9-ylamino)-heptyl]-benzamide (2f)

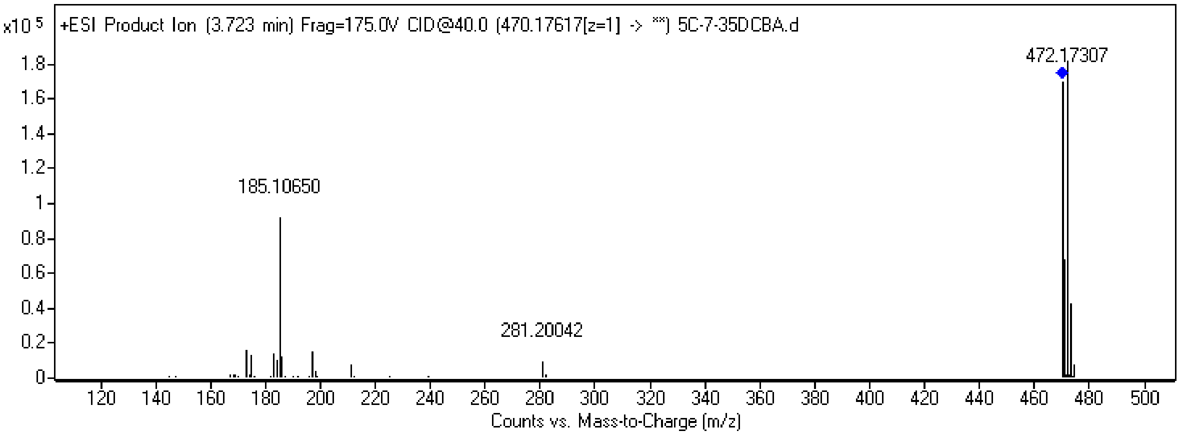

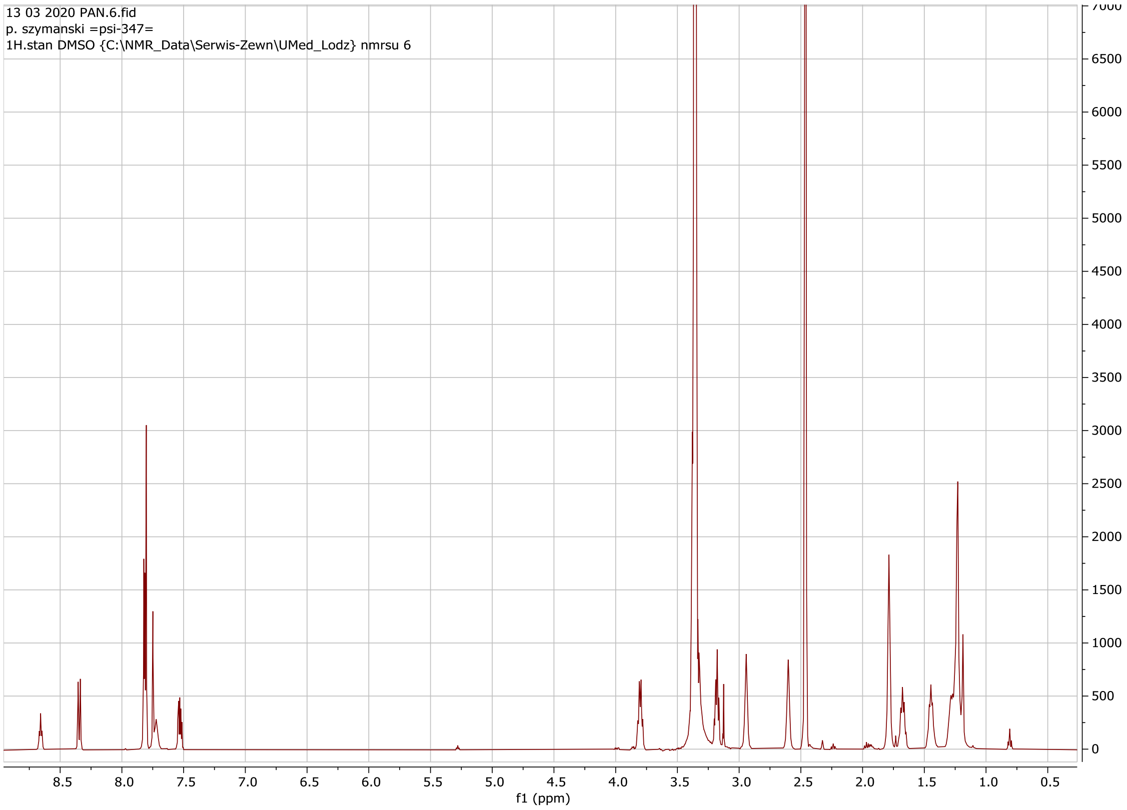

3,5-Dichloro-N-[8-(2,3-dihydro-1H-cyclopenta[b]quinolin-9-ylamino)-octyl]-benzamide (2g)

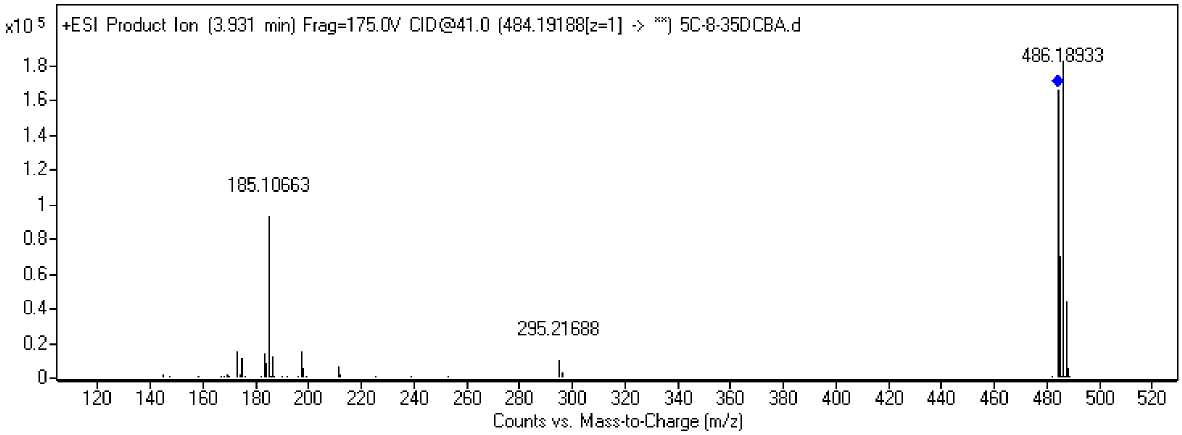

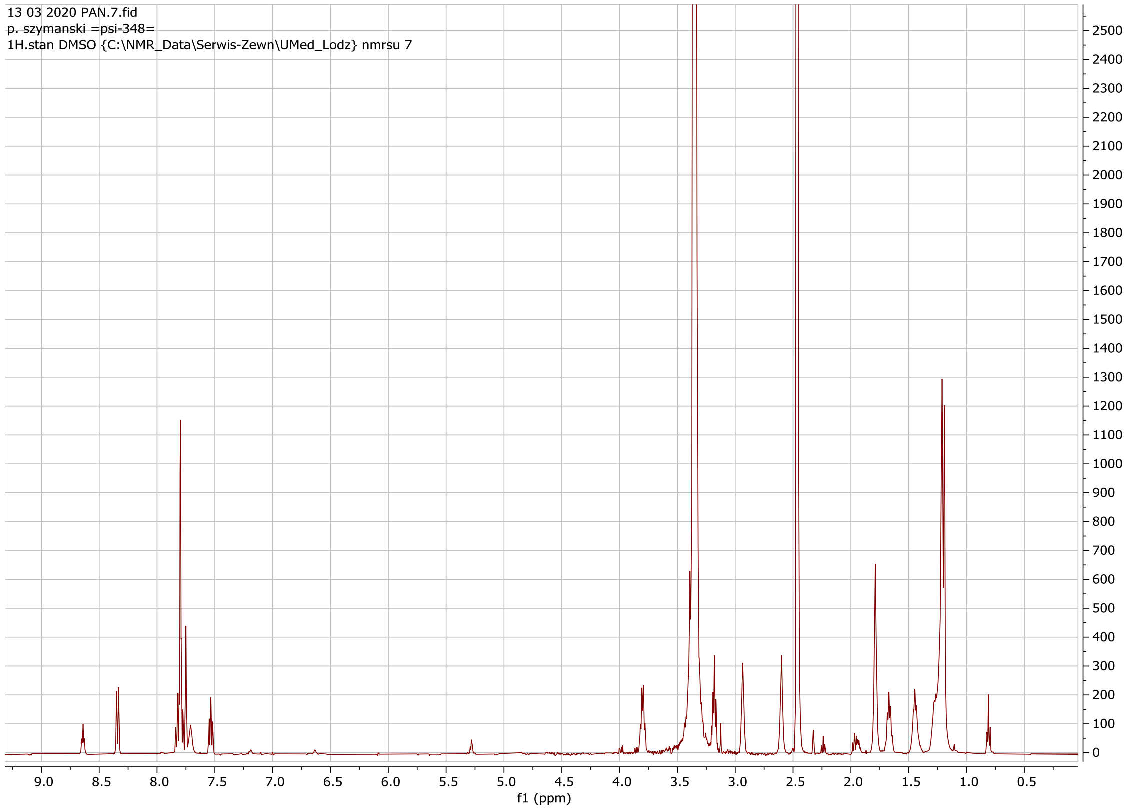

3,5-Dichloro-N-[9-(2,3-dihydro-1H-cyclopenta[b]quinolin-9-ylamino)-nonyl]-benzamide (2h)

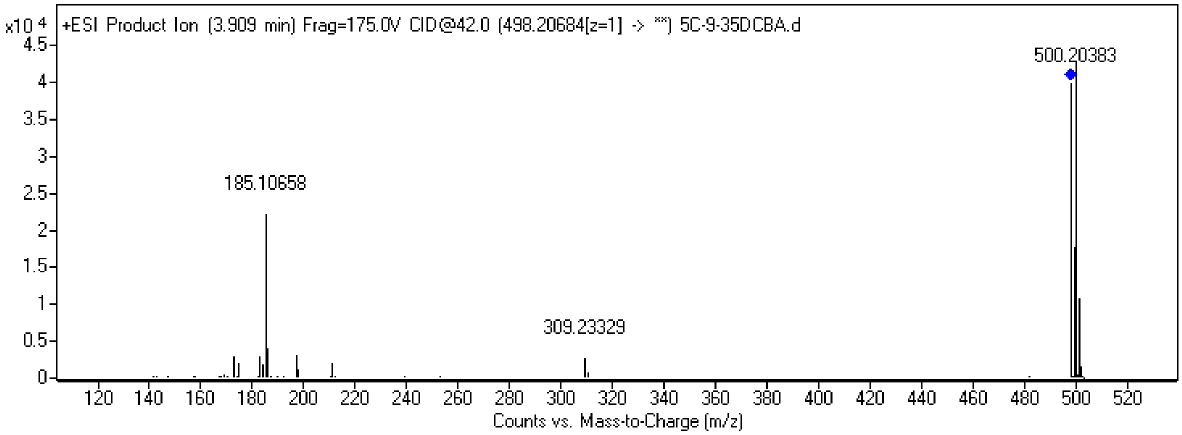

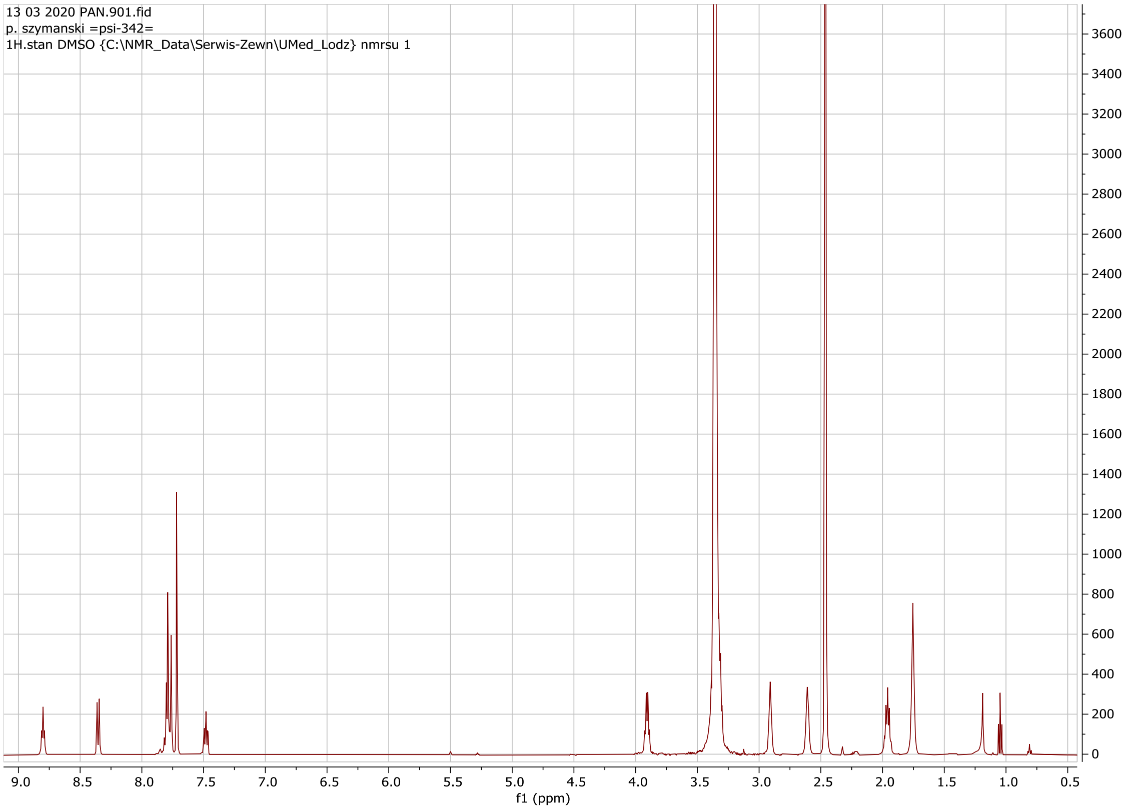

MS spectra of compounds 3a-3h

3,5-Dichloro-N-[2-(2,3-dihydro-1H-cyclopenta[b]quinolin-9-ylamino)-ethyl]-benzamide hydrochloride (3a)

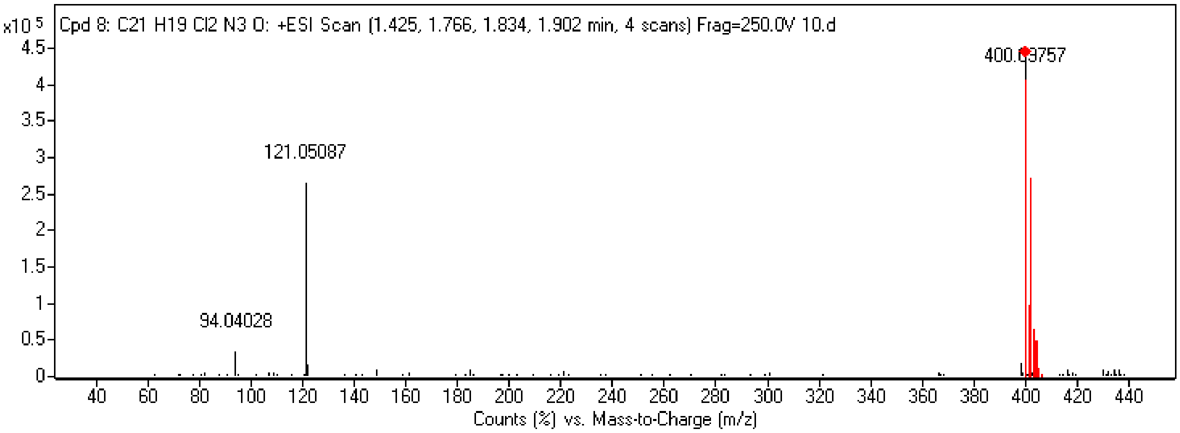

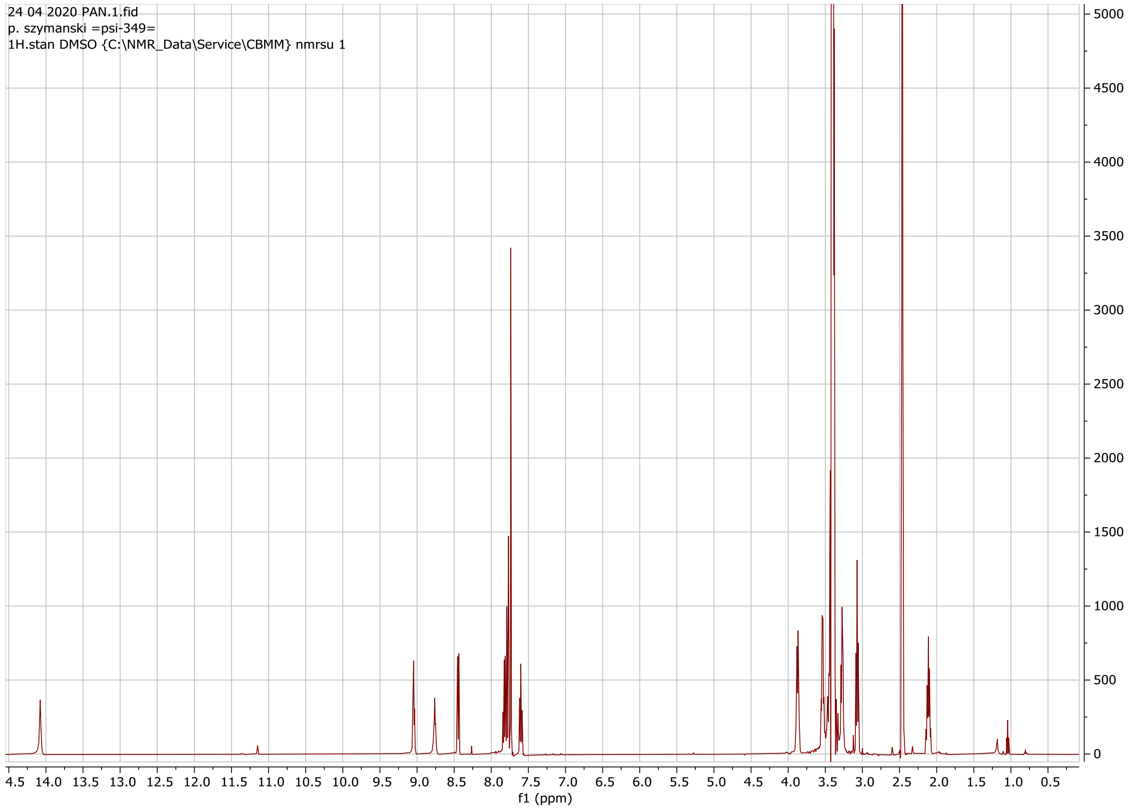

3,5-Dichloro-N-[3-(2,3-dihydro-1H-cyclopenta[b]quinolin-9-ylamino)-propyl]-benzamide hydrochloride (3b)

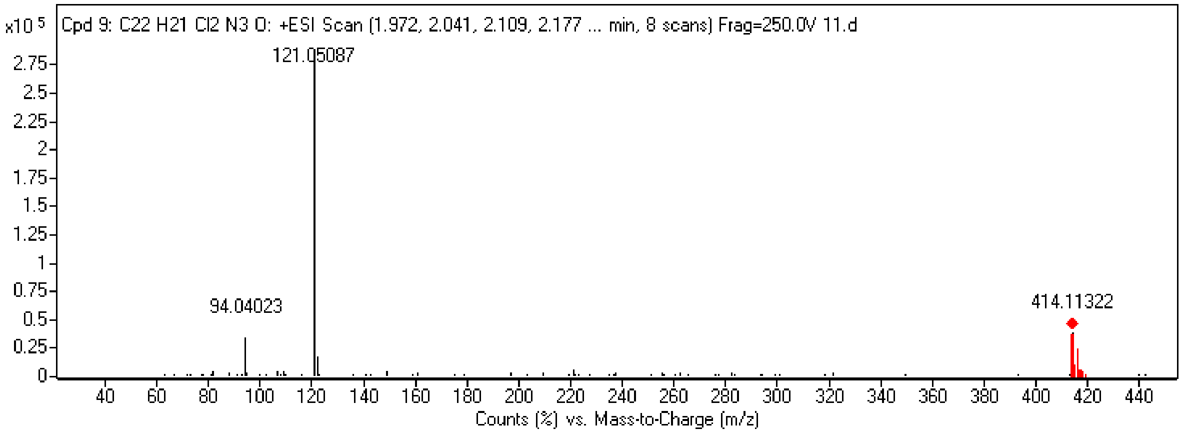

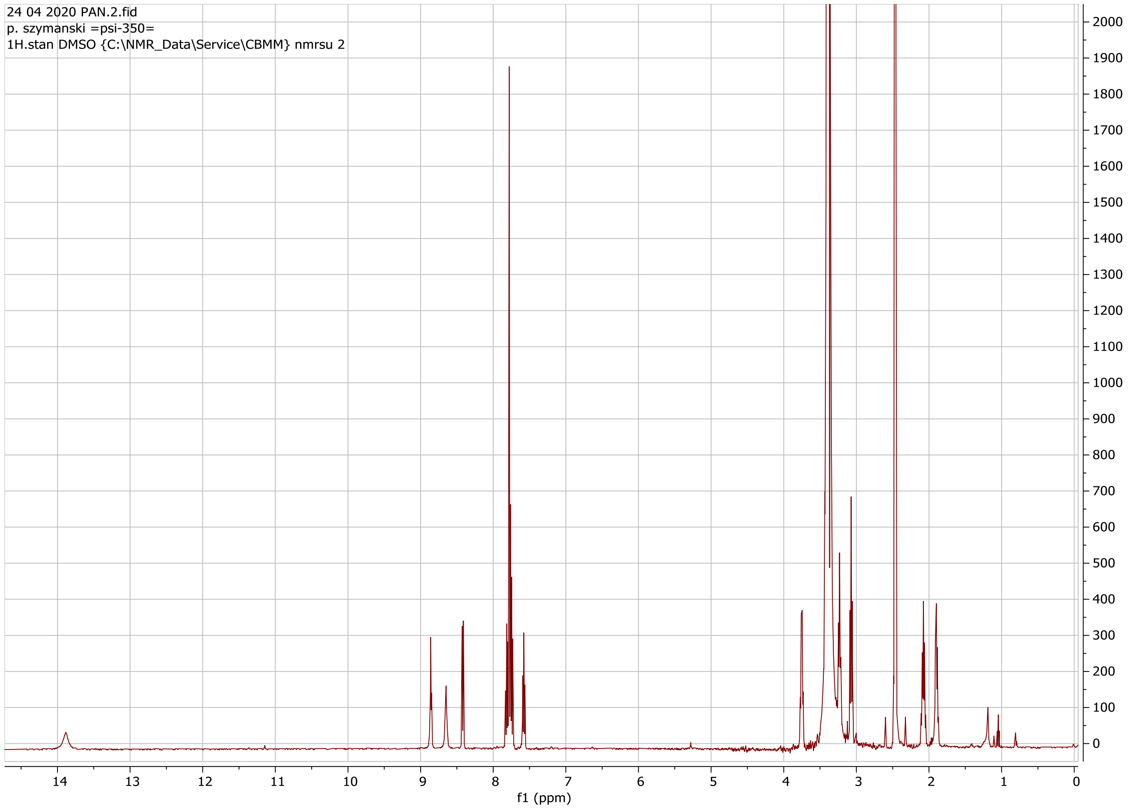

3,5-Dichloro-N-[4-(2,3-dihydro-1H-cyclopenta[b]quinolin-9-ylamino)-butyl]-benzamide hydrochloride (3c)

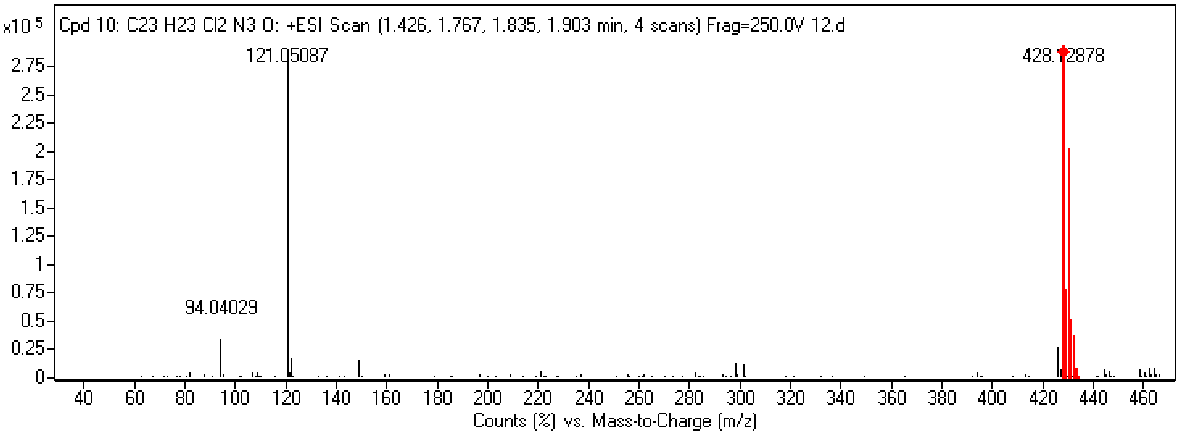

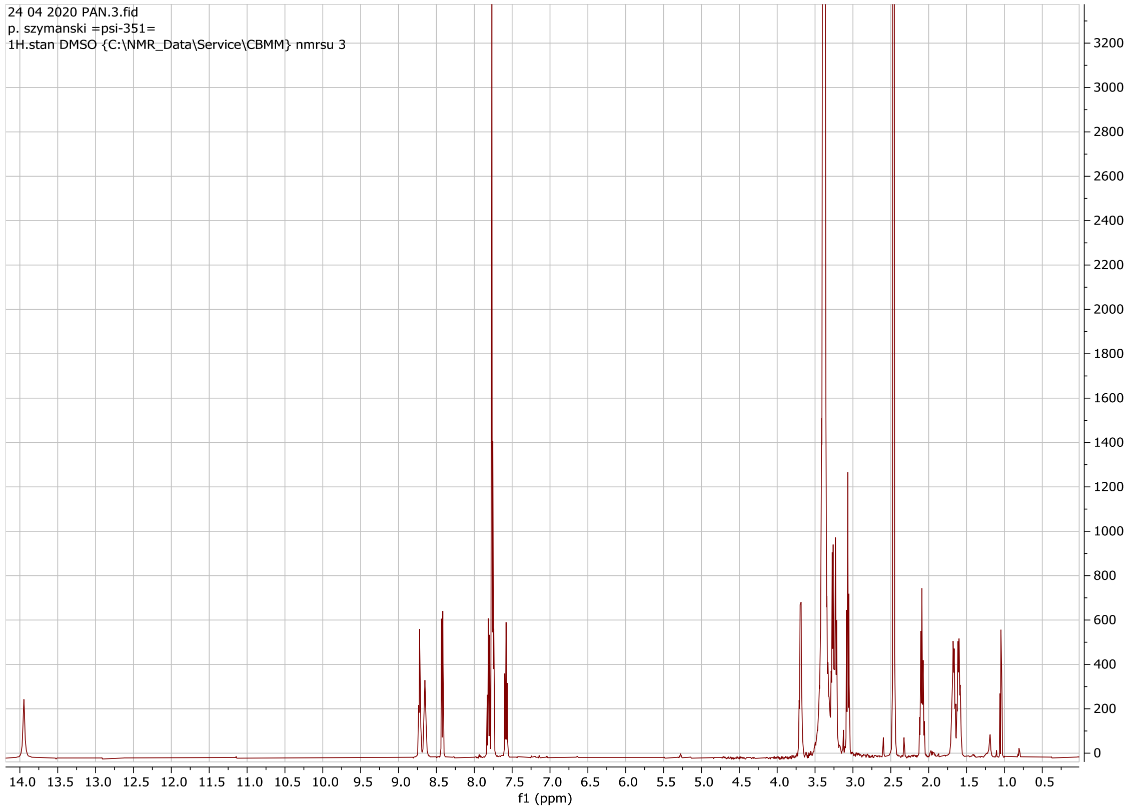

3,5-Dichloro-N-[5-(2,3-dihydro-1H-cyclopenta[b]quinolin-9-ylamino)-pentyl]-benzamide hydrochloride (3d)

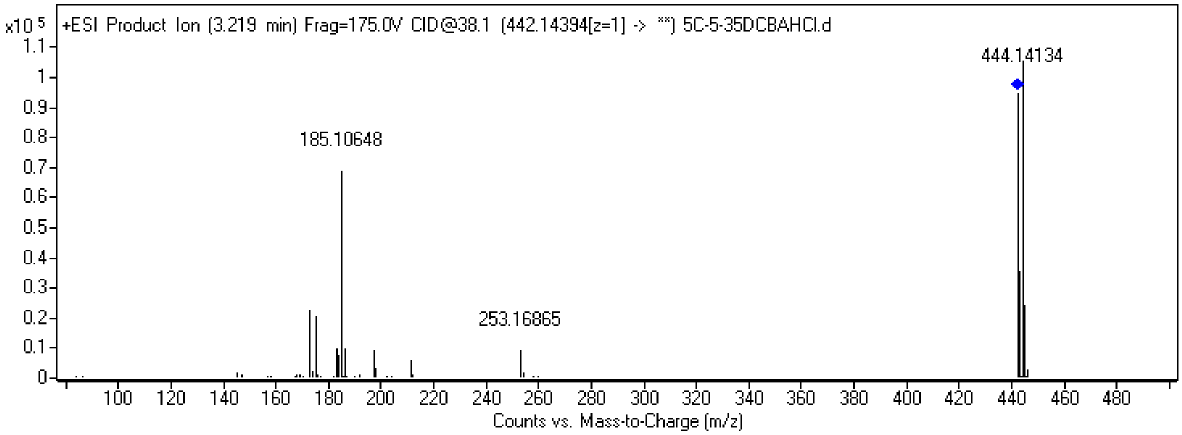

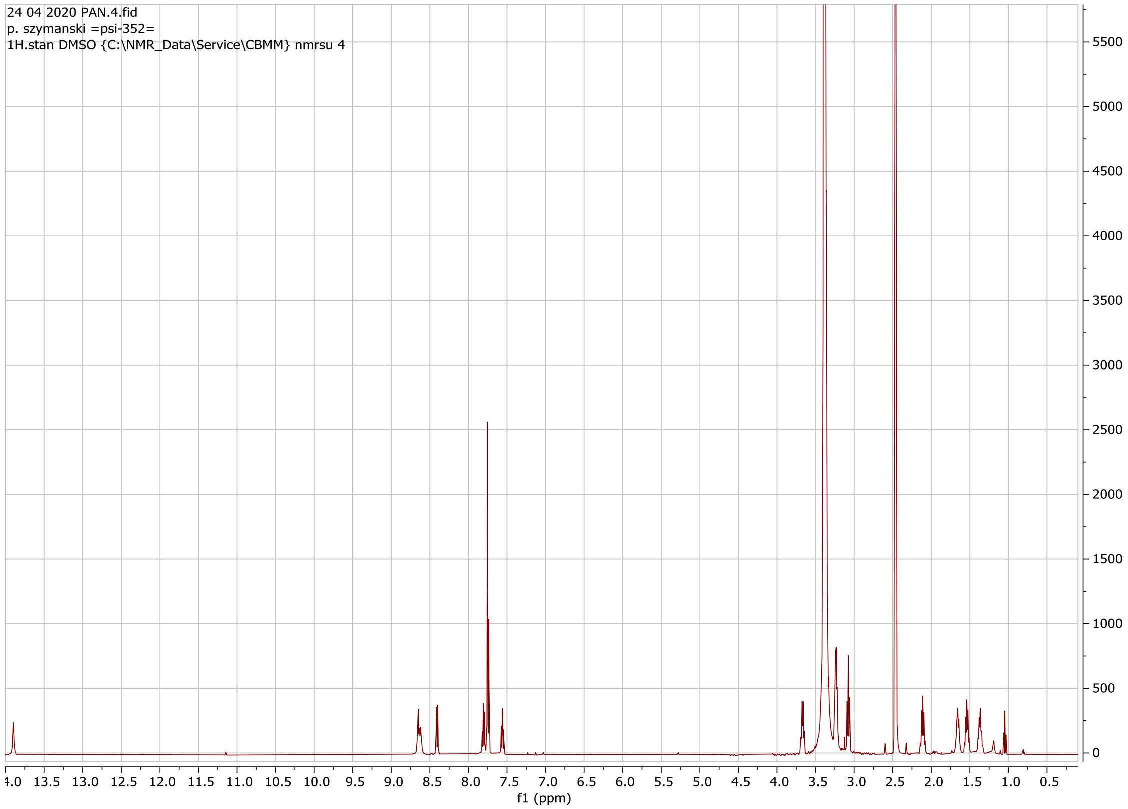

3,5-Dichloro-N-[6-(2,3-dihydro-1H-cyclopenta[b]quinolin-9-ylamino)-hexyl]-benzamide hydrochloride (3e)

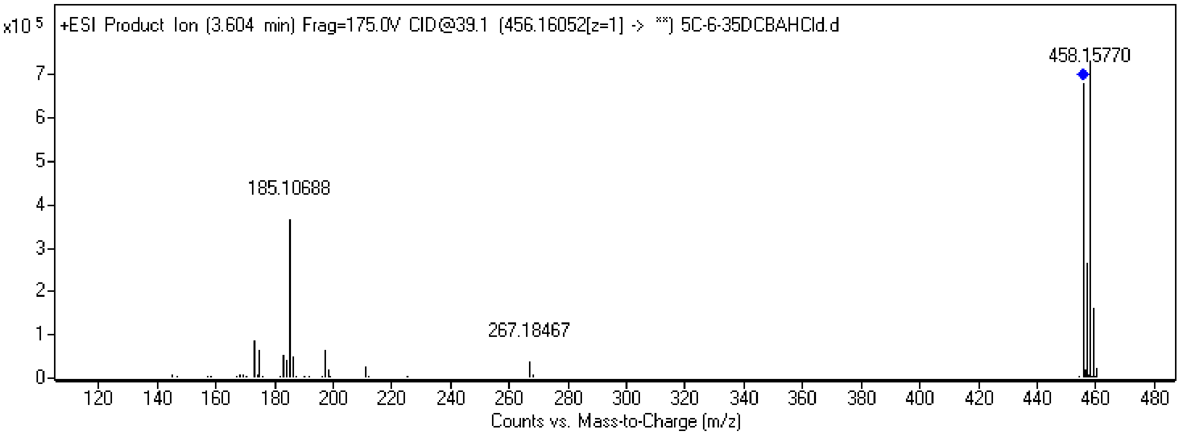

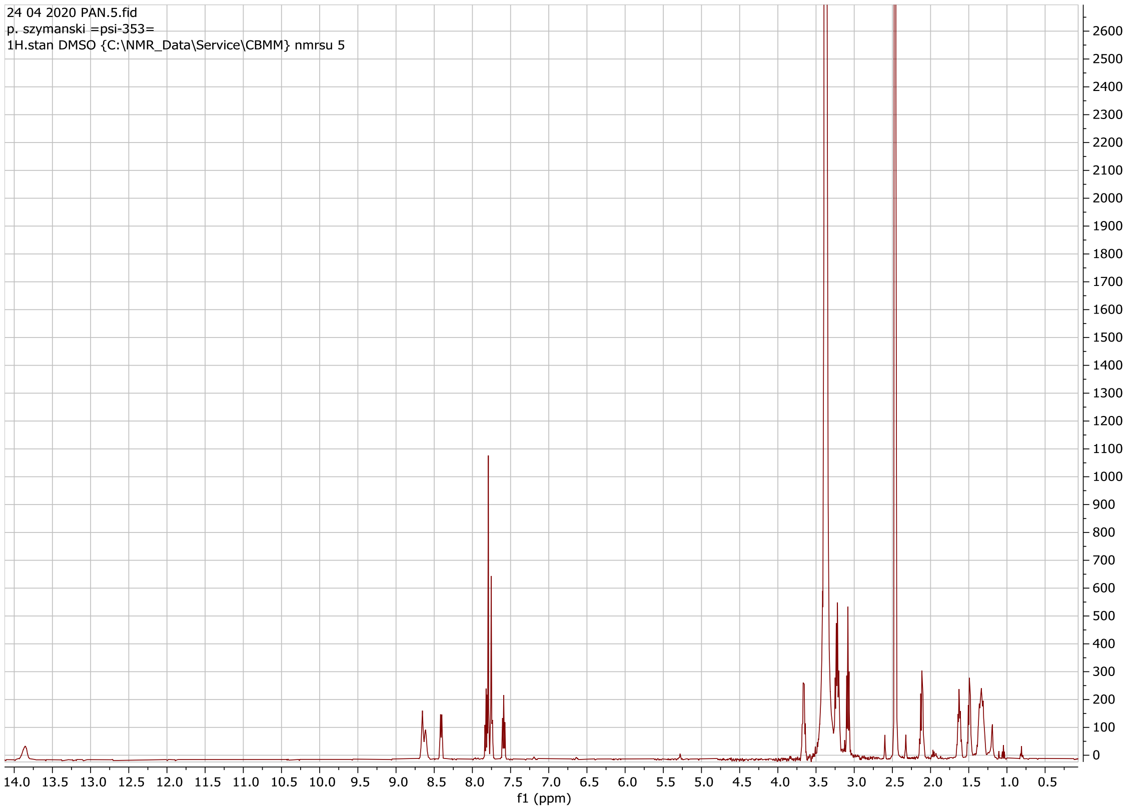

3,5-Dichloro-N-[7-(2,3-dihydro-1H-cyclopenta[b]quinolin-9-ylamino)-heptyl]-benzamide hydrochloride (3f)

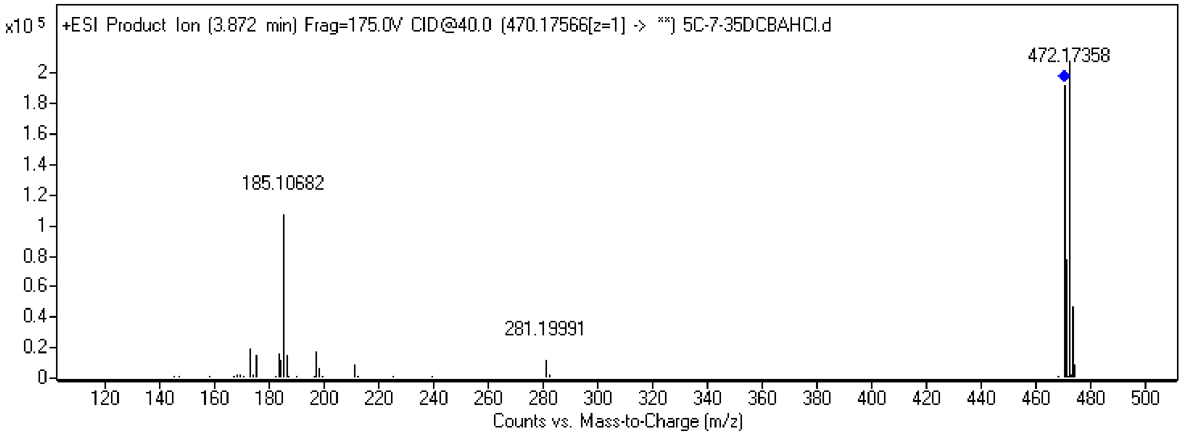

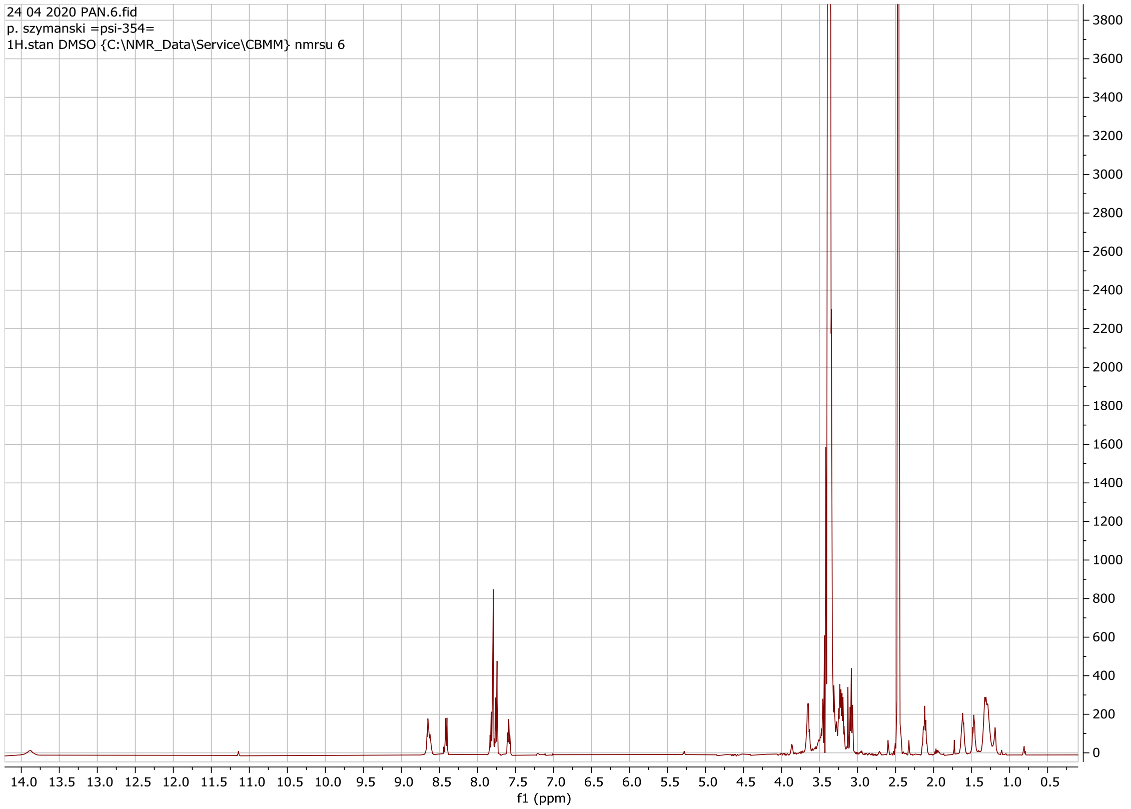

3,5-Dichloro-N-[8-(2,3-dihydro-1H-cyclopenta[b]quinolin-9-ylamino)-octyl]-benzamide hydrochloride (3g)

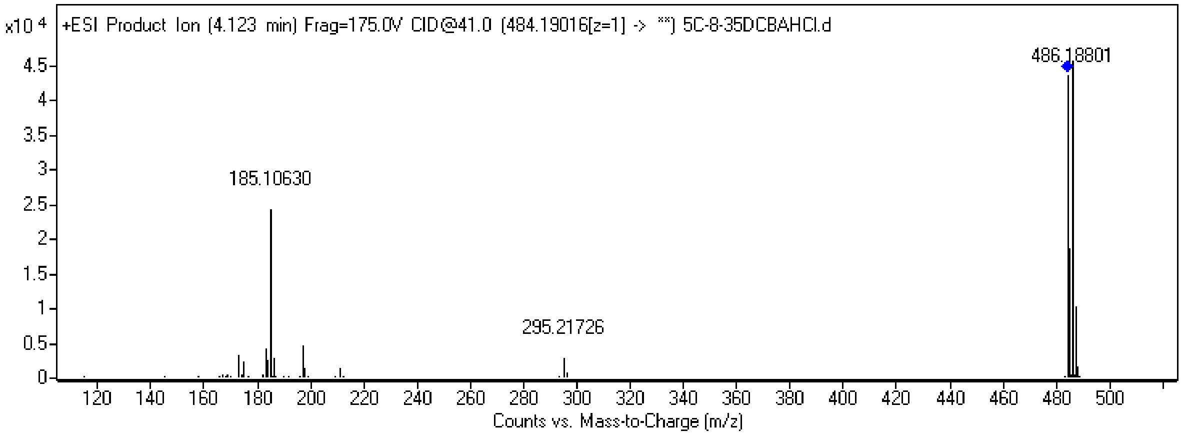

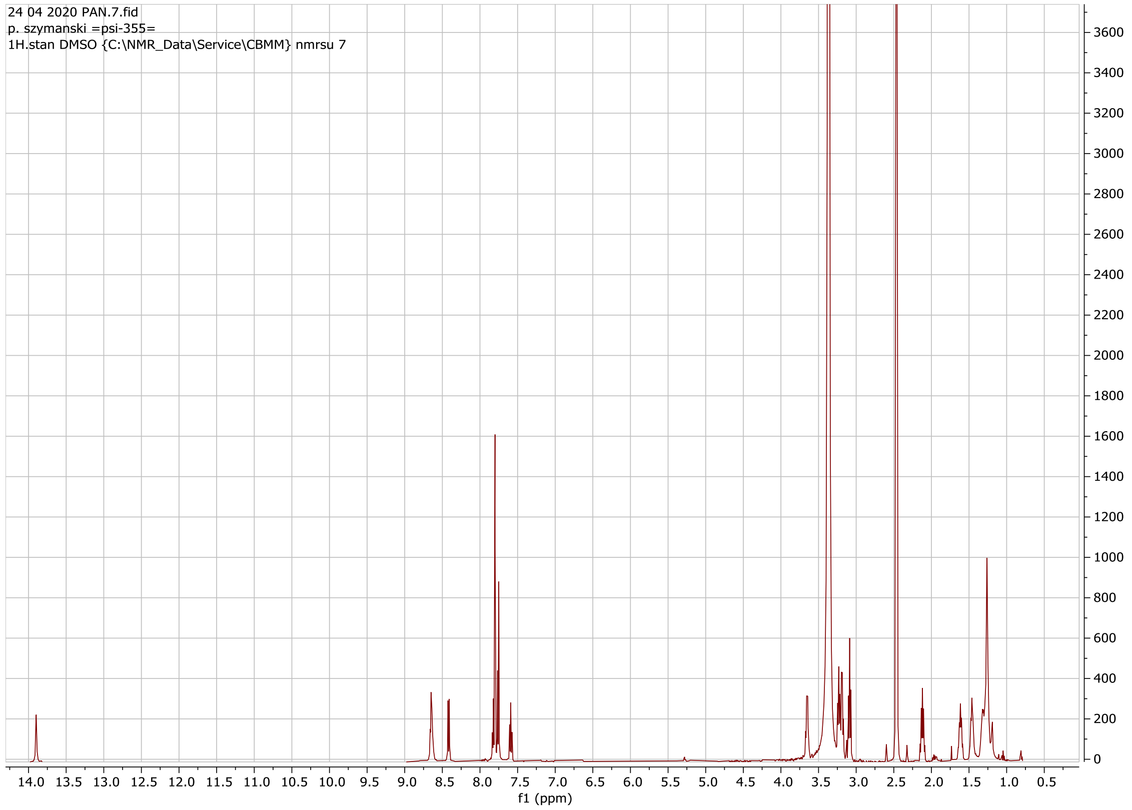

3,5-Dichloro-N-[9-(2,3-dihydro-1H-cyclopenta[b]quinolin-9-ylamino)-nonyl]-benzamide hydrochloride (3h)

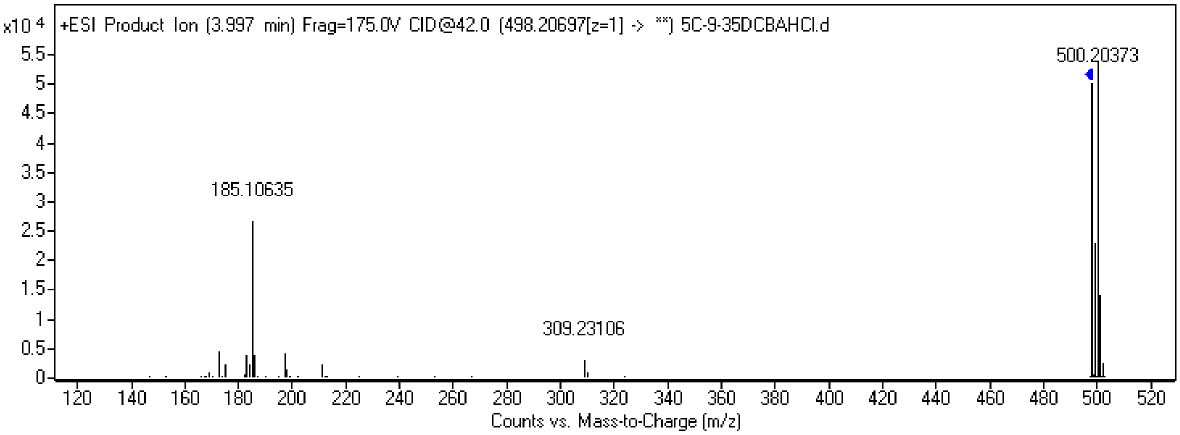

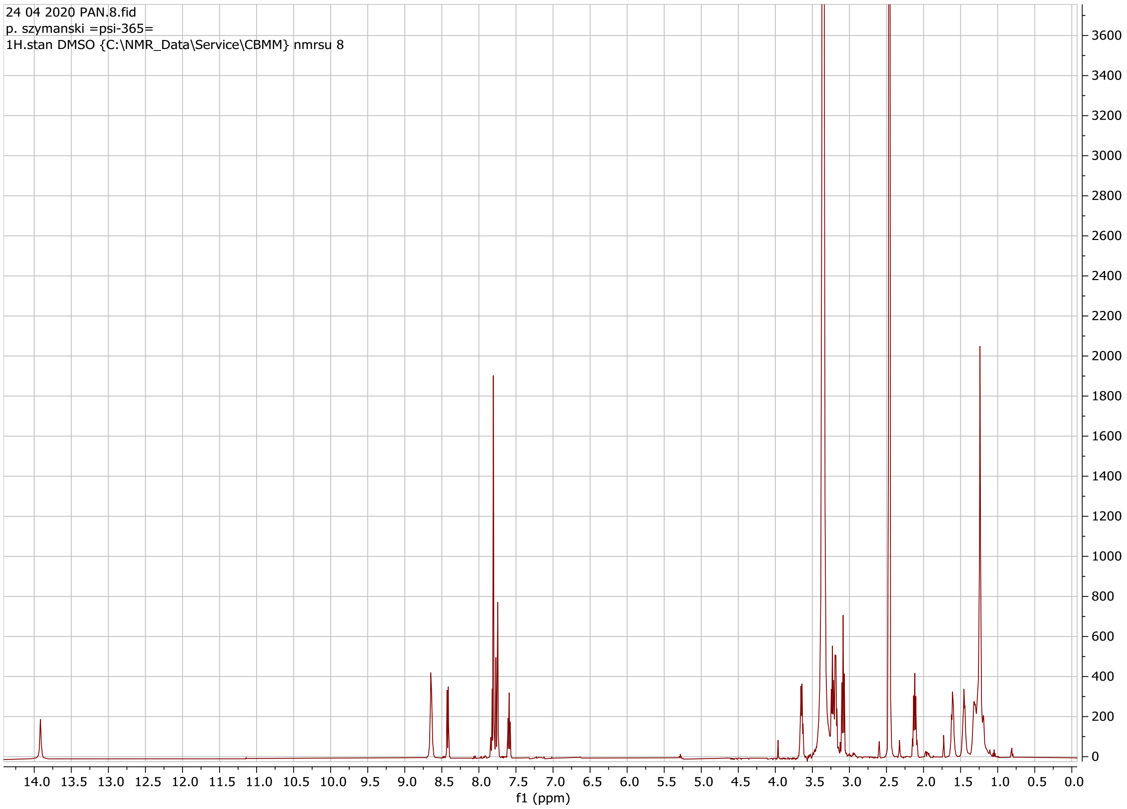

Supplement: Supplemental Material [file IENZ_A_2158822_SM9482.pdf]
